# Fault Tolerance Structures in Wireless Sensor Networks (WSNs): Survey, Classification, and Future Directions

**DOI:** 10.3390/s22166041

**Published:** 2022-08-12

**Authors:** Ghaihab Hassan Adday, Shamala K. Subramaniam, Zuriati Ahmad Zukarnain, Normalia Samian

**Affiliations:** 1 Department of Communication Technology and Network, Faculty of Computer Science and Information Technology, University Putra Malaysia, Serdang 43400, Malaysia; 2 Computer Science Department, Faculty of Computer Science and Information System, University of Basrah, Basrah 61004, Iraq

**Keywords:** Wireless Sensor Networks (WSNs), Fault Tolerance (FT), error detection, error diagnosis, error recovery

## Abstract

The Industrial Revolution 4.0 (IR 4.0) has drastically impacted how the world operates. The Internet of Things (IoT), encompassed significantly by the Wireless Sensor Networks (WSNs), is an important subsection component of the IR 4.0. WSNs are a good demonstration of an ambient intelligence vision, in which the environment becomes intelligent and aware of its surroundings. WSN has unique features which create its own distinct network attributes and is deployed widely for critical real-time applications that require stringent prerequisites when dealing with faults to ensure the avoidance and tolerance management of catastrophic outcomes. Thus, the respective underlying Fault Tolerance (FT) structure is a critical requirement that needs to be considered when designing any algorithm in WSNs. Moreover, with the exponential evolution of IoT systems, substantial enhancements of current FT mechanisms will ensure that the system constantly provides high network reliability and integrity. Fault tolerance structures contain three fundamental stages: error detection, error diagnosis, and error recovery. The emergence of analytics and the depth of harnessing it has led to the development of new fault-tolerant structures and strategies based on artificial intelligence and cloud-based. This survey provides an elaborate classification and analysis of fault tolerance structures and their essential components and categorizes errors from several perspectives. Subsequently, an extensive analysis of existing fault tolerance techniques based on eight constraints is presented. Many prior studies have provided classifications for fault tolerance systems. However, this research has enhanced these reviews by proposing an extensively enhanced categorization that depends on the new and additional metrics which include the number of sensor nodes engaged, the overall fault-tolerant approach performance, and the placement of the principal algorithm responsible for eliminating network errors. A new taxonomy of comparison that also extensively reviews previous surveys and state-of-the-art scientific articles based on different factors is discussed and provides the basis for the proposed open issues.

## 1. Introduction

The exponential growth of the Industry Revolution 4.0 (IR4.0) concept is a fundamental paradigm that encompasses, among other future industrial innovations, the IoT, the Internet of Services (IoS), and WSNs [1]. WSN is the backbone of the IoT architecture, which allows us to detect our surroundings, collect vital statistics, and send them to the final destination called the Base Station (BS) [2]. Therefore, the IoT is highly oriented toward the WSN as a critical platform for data sensing and communication [3]. WSN offers the infrastructure for the evolving IoT involving a wide spectrum of areas and fields [4]. For example, intelligent products such as sensor devices, camera systems, and wearable technology are used in a wide range of situations such as intelligent homes and intelligent transportation. Smart items are also used in various other applications such as agriculture, smart communities, innovative medical services, and military applications [5,6]. 

WSNs have become more prevalent in wireless communication, generally described as multi-hop network systems composed of a broad variety of smart sensor nodes. These nodes consisting of dual roles, which are sensing and routing, have attributes that are distributed auto-organizing and energy-constrained [7]. Each sensor node has the ability to collect data from the environment required for a particular application and can also function as a data forwarder to ensure that the data have reached their final destination. The independent and versatile nature of WSN operations is always desired in many critical and real-time application scenarios, such as earthquake and glacial movement monitoring, volcano activities monitoring, forest fire monitoring, and so on [8].

The expected number of sensors that will be deployed around the world is projected to reach one trillion by 2025 [9]. Consequently, an enormously high volume of data are being collected from a highly diversified and wide range of WSNs [10]. Thus, parallel to this rise in diversity and importance is the constant pressing need to address and provide dynamic solutions. Prerequisites of data integrity, data accuracy, and data reliability are compulsory, especially when dealing with hazardous environments [11]. Fault tolerance is the network’s ability to proffer a desirable and required degree of functionality and reliable data when faults are present [2]. FT is one of the essential requirements to be constantly monitored and adhered to in WSNs due to the high probability of hardware failures such as sensor failure, link failure, and malicious attacks. As long as the WSN is an error-prone network [12], well-organized fault detection is needed to locate the errors which occur in the network. Fault tolerance structure consists of fault identification, diagnosis, and correction methods [13]. FT is a crucial aspect of WSN, and it is important to embed the network with a technique for detecting measurement errors before this incorrect data reaches the BS. 

FT is also correlated to the primary notion of network reliability [14]. Therefore, the fault-tolerance must satisfy two main clauses. First, faults will produce erroneous readings that may pose a high risk in certain situations before and when they reach the BS. Second, these fault readings consume a huge amount of energy due to the meaningless and additional high cost associated with the respective sending operations. In addressing these attributes many routing protocols in WSNs contain built-in techniques for differentiating between a real event and a measurement fault [15]. The spectrum of strategies to address these attributes has provided a rich and heterogeneous repository of routing algorithms. In relation to providing the analytics to extract the distinction in each of them, this study has the following objectives to be met:

- A comprehensive and extensively analyzed literature survey of the latest and fundamentally critical studies that address in detail fault tolerance approaches in WSNs.
- A new taxonomy that provides a comprehensive classification for fault tolerance techniques for research in this area was conducted within a significant time frame that has not been previously addressed in an extensive manner, which is 2016–May 2022.
- To identify and discuss the open issues deduced from the proposed taxonomy of comparison and the enhanced fault tolerance management architecture needed in WSN.

The remainder of this paper is organized into eight sections. Section 2 discusses in detail the state-of-the-art survey and motivation of fault tolerance classification methods. Fault classifications will be addressed in Section 3. The main aspects of FT structures are reviewed in Section 4. Proposed performance metrics within the FT techniques are presented in Section 5. In Section 6, a proposed classification of fault tolerance approaches is presented. Comparative analysis and detail discussions of the FT approach in WSNs are presented in Section 7. Open research issues are discussed extensively in Section 8. The conclusion is drawn in Section 9.

## 2. State of the Art Surveys and Motivation of Fault Tolerance Classification Methods

Fault tolerance is crucial in WSNs due to the high need for reliable and integral data that will be produced from the network, no matter which kind of application this network serves [16]. Three main digital databases have been chosen and searched to increase the odds of getting the best search results, which are: (1) Science Direct (SD), which provides access to a variety of journals covering a range of scientific disciplines, including science and technology; (2) IEEE Xplore, a digital library of engineering and technology publications; and (3) Scopus, which provides access to numerous articles covering a range of disciplines. These databases were chosen based on their academic qualifications and presentations in a variety of academic disciplines. The first revision step began with the selection of an estimated (*n* = 4954) publications from these three databases. After selecting articles from the range of the previous six years, a total of (*n* = 2413) were obtained. The third screening stage was the scanning of titles and abstracts, which yielded a total of (*n* = 242) articles. The last filtration process involved examining the whole text of recognized articles from the previous phase and after the duplicate screening was completed. Based on our criteria, a total of (*n* = 62) publications were reviewed and has been judged to be relevant to this review. These scientific studies have drawn the focus to research work from 2016 to May 2022. The research works within the mentioned timeframe were chosen carefully to provide a new survey that differs from previous surveys especially in encompassing the trends acquired from the scientific content. Moreover, our review has also ensured the inclusion of eighteen (18) review articles represented in Table 1 and Table 2. These surveys represent the substantial surveys on the topic since 2007.

Table 1 and Table 2 show that a substantial number of surveys have been done in this area, providing distinct classifications [17,23]. However, analyzing these surveys in detail has enabled several open issues pertaining to these surveys which are deliberated as follows:

1. The coverage of the articles is on certain specific areas of algorithms and classifications.
2. The time scale of the related works under examination is within a specific time period that creates its respective constraints of future applicability.
3. The absence or the duplication of state-of-the-art open issues related to fault tolerance in WSN.
4. Many previous studies were related to a specific type of the WSN concept, such as Mobile Ad Hoc Network (MANET), Flying Ad Hoc Network (FANET), and Underwater Sensor Network (USN).
5. Many studies were on a specific type of fault tolerance, such as fault tolerance via clustering approaches, fuzzy approaches, or statistical approaches.

As a result, with the continued exponential growth of WSN and the paradigm changes of operating prerequisites there is a definite multifold benefit in presenting a more comprehensive and state-of-the-art review. The pressing need is to give an exhaustive examination and analysis of all the modern methods for fault tolerance that work in WSNs. The detailed review and analysis will address different open issues and carefully selected performance metrics that distinctly complement but differ from other survey articles. Thus, this paper’s key goals are as follows:

⬝ classifying fault in a sensor network based on new metrics, which are fault pattern and stability, network components, and fault-affected area.
⬝ categorizing faults into five classes based on: behavior, time, components, the affected area, and layers.
⬝ categorization of fault-tolerant components into three vital stages of the fault tolerance architecture which are: error detection, error diagnosis, and error recovery.
⬝ proposing a new taxonomy for fault tolerance structure that encompasses general classes and subclasses based on their performance.
⬝ defining the existing fault tolerance approaches and analyzing the most important steps in error detection, error diagnosis, and error recovery.

The primary goal of this survey is to respond to some pertinent questions which are stated as follows:

1. What are the most critical faults impacting WSNs that need to be addressed?
2. When it comes to WSNs, what are the basic fault management procedures?
3. What are the main operations for each stage in WSN?
4. What methods may provide a thorough classification for fault tolerance structure?
5. What are the most significant difficulties associated with fault management?
6. Are there any fault tolerance systems that need to be estimated or evaluated?
7. Will fault management methods evolve, embracing new paradigms such as artificial intelligence (AI) and other features in the future?

## 3. Faults Classifications in WSN

**Figure 1:**
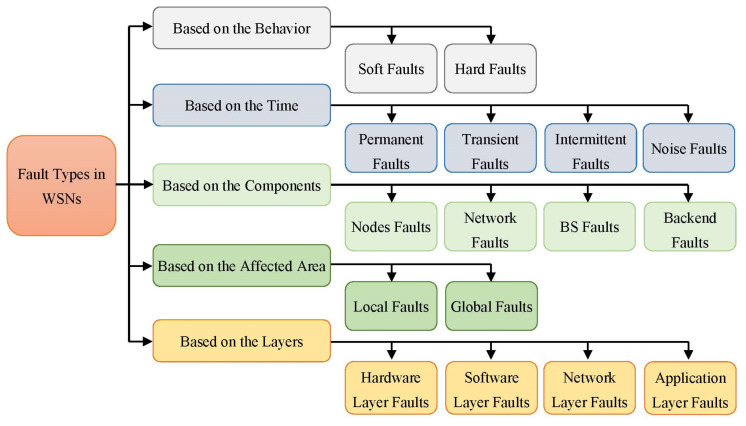
A taxonomy for the different fault types in WSNs.

During the last years, different classifications of faults have been proposed in WSNs [32,35,36,37]. A clear understanding of these various classifications provides a defined foundation and enhancements to the proposed algorithms developed to address fault-related issues. Figure 1 illustrates the various categories of errors in WSN as deliberated, respectively, in [17,21,24]. Node behavioral faults, fault period, network infrastructure elements, the region impacted by a fault, and the layer where the error occurred are all factors considered in determining the overall categories [36,37].

The remainder of this section explains in detail the general classification of faults in WSNs. Faults can be classified, depending on the behavior-base, into two types of errors. A hard fault happens once a sensor node is unable to connect with other nodes due to module failure for example the case of a dead node owing to energy depletion, while soft faults occur when sensor nodes continue to function and communicate through other sensor nodes but they sense, process, or send incorrect data [38,39]. 

Permanent, transient, intermittent, and noisy are the four types depending on the duration of the failures. Permanent faults are long-lasting and persistent. A faulty battery, for example, is an example of a permanent fault. On the other hand, the failure may temporarily affect the node. Transient faults are not permanent or continuous; they may develop due to transient environmental changes. They appear briefly and then disappear, although they may reappear. Diagnosing and handling transitory problems is very challenging [40].

Unlike transitory errors, intermittent errors occur over an extensive length of period. They may occur at irregular intervals and with a predictable frequency; they are easy to detect and treat [41]. When there are noise errors, the sensor values become more variable. Noise faults impact a series of sensor node interpretations, unlike transient faults, which disturb one sensor node reading at a time [42].

Another type is based on network components: node, network, BS, and backend faults [43]. The node failure is so popular in WSNs because the node plays a significant role in the network. Two main reasons cause node errors. Firstly, hardware errors include microcontroller failures, sensing unit failures, memory failures, and battery failures [21,24]. Secondly, software errors have routing failures, Media Access Control (MAC) failures, and application failures. In general, node failures result in erroneous network judgments, particularly when the failures are linked to cluster heads. When incorrect data are collected, and inaccurate information is delivered to the BS, improper information will be from the whole network. As a result, the majority of research focuses on failure detection and recovery in sensor nodes, particularly cluster heads, master nodes, and backbone nodes.

One of the most serious network flaws is routing process failure, which may result in the transmission of erroneous data or excessive delays [16]. Because all networks are prone to a connection failure, unstable relationships between nodes result in network separation and dynamic changes in network topology. Network failures include radio interference, path faults, permanent or temporary path blockages, and simultaneous transmission. The data are sent to the backend system via the BS. This section may include errors resulting in the loss of network-wide data. For example, a problem with the BS may prohibit duties from being sent to sensors. Furthermore, congestion in a local region may extend to the BS, affecting data reception from other areas of the network [30]. The lack of energy in this part of the network is one of the serious faults. Because BS is often situated distant from cities, it has limited and restricted energy and is prone to developing errors. Furthermore, the software utilized in BS may develop faults.

Lastly, the data collected in the BS is examined and assessed in the backend faults. Hackers may cause backend errors, resulting in defective nodes and network failure [30,31]. This failure impacts the whole network, resulting in system inefficiencies. Brief descriptions of faults are categorized according to their area of effect. A local fault occurs when a fault impacts one or more nodes. Nevertheless, some key nodes, such as the cluster head, backbone node, or manager node, have known issues regarded as global faults. Disregarding efforts to correct local problems creates global errors. For example, errors in sensor nodes lead to erroneous data being delivered to the BS.

Another perspective is that the faults are broken down into four types based on the layers in which the errors have occurred [23]. Hardware layer errors are the first type in this classification. The quality of the node’s component, the restricted power resource, and the harsh environment are some examples of hardware faults in WSNs. Hence, faults in this layer are malfunction caused by one or some node components. Software layer errors are the second class that is represented by two parts. The system’s software, such as the operating system, and the system’s middleware, such as the routing and aggregation procedures. Network layer errors are the third type of fault in WSNs. The network layer is crucial because the wireless links are prone to failure in every wireless network. The errors in this layer are caused by the harsh environment and interference phenomena among the nodes [44]. 

Application layer errors are the fourth and last type in this taxonomy. Each application has its own set of faults that are distinct from those of the other applications. The most frequently encountered errors at the application layer relate to coverage and connectivity.

In conclusion, WSN is described as a network prone to failure, with many error types within it. Therefore, it is compulsory to have a complete fault tolerance structure to minimize the effect of these errors. The next section clarifies the concept of fault tolerance and its main structure that deal with faults in any WSN.

## 4. The Main Aspects of Faults Management Structure in WSNs

FT refers to a system’s ability to handle mistakes while still delivering its optimal performance [24,32]. The result of a combination of fault detection, diagnosis, and repair is fault tolerance. It is a significant problem in WSN applications for delivering trustworthy data. It should guarantee that a system is available for usage during a duration of a failure or disruption. Therefore, fault tolerance improves the WSN structure’s availability, reliability, and dependability [45]. It is necessary to review a summary of the three major principles of fault tolerance management structures. Fault management is one of the most popular methods for increasing fault tolerance [46]. 

**Figure 2:**
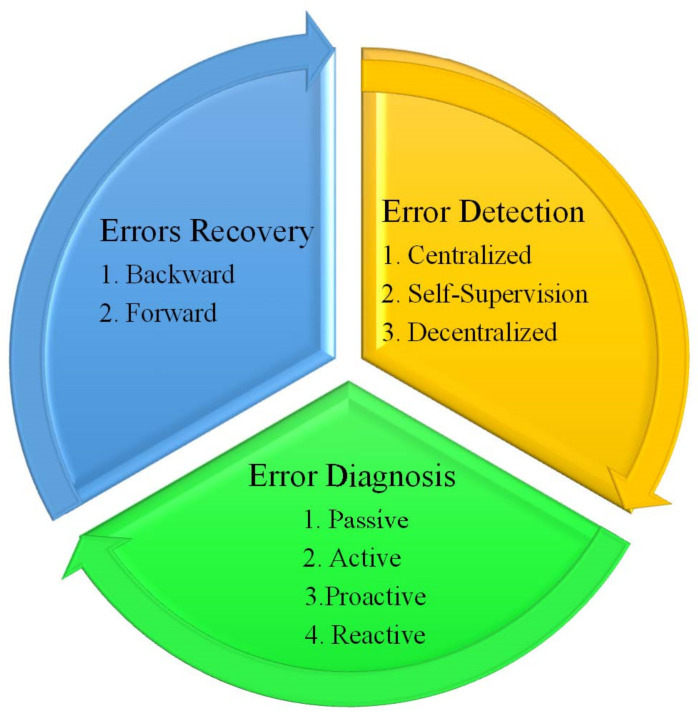
General steps for fault tolerance structure in WSN.

As previously stated, the fault management structure in WSNs consists of three stages: error detection, diagnosis, and recovery as shown in Figure 2. The following subsections describe the three phases of the fault management framework.

### 4.1. Error Detection

Error or fault detection refers to identifying any unexpected failure or damaging forces that affect a network’s or node’s optimum condition [47]. Based on their performance, fault detection methods are divided into three categories: centralized, self-supervision, and decentralized [19,31,34]. They will be addressed further down.

A sensor node detects problems centrally in a centralized error detection method [48]. The central node in this approach gets status messages from other nodes regularly and uses them to identify problematic nodes [49]. The central node in this approach gets status messages from other nodes regularly and uses them to identify problematic nodes [35]. In addition, as the number of nodes grows, so does the number of messages deliver to the center. As a result, detection latency increases, making the technique unsuitable for use in real-time settings [41]. As a result, centralized techniques cannot be used in every WSN. In addition, a method known as self-supervision is used, whereby a sensor node examines and evaluates its abilities and physical conditions. In addition, sensor nodes monitor the remaining energy of their batteries and estimate the battery’s lifespan by studying and calculating the amount of time and rate at which the battery is discharged. This technique has a low detection latency, and it is scalable. However, since the focus of self-supervision techniques is on persistent defects in nodes, they cannot identify all errors in a network [25]. The use of exact assumptions and threshold values is needed in self-supervision methods; however, it is not feasible to acquire these values in some WSNs due to technical limitations.

The goal is to include all nodes in the detecting process in the decentralized (distributed) method [19,21]. Faults are identified in this method via the cooperation of adjacent nodes and the use of clustering algorithms, respectively. When using the former method, data from neighbors are used in conjunction with particular techniques, for instance, majority vote or analyzing the information obtained with the average of the information received from neighbors [27]. Cluster Heads (CHs) are used to identify problems in clustering techniques. Because cluster heads may become inaccessible when faults arise, the detection of defective clusters has piqued the attention of researchers worldwide. Nowadays, strategies for decentralized fault management are gaining popularity [19,25]. The accuracy of defect detection improves as the number of nodes involved increases. However, updated data from neighbors are required to identify errors with neighbor-cooperation-based techniques. In addition, as the number of participating nodes grows, so does the amount of control messages transmitted across the network, resulting in increased energy usage and congestion. Cluster-based techniques aim to increase the scalability and reduce the amount of energy consumed, whereas detection latency methods are the most compatible in WSNs [16].

### 4.2. Error Diagnosis

To properly fulfill the fault-tolerance principle, it is necessary to identify the kind of error and remark faulty nodes. The source, nature, and impact of failures on the network’s status should all be determined [50]. One well-known approach is to use specific reference nodes in a network with particular geographical positions to assist other nodes in locating their location. The need to monitor the WSN is raised to investigate and locate network errors. Monitoring may be divided into four categories: passive, active, proactive, and reactive. They will be discussed and judged further below. The passive model triggers alerts to notify the BS whenever a fault is discovered in the passive monitoring model [51]. Because the technique does not need the transmission of consecutive messages to assess the network, it consumes less energy and generates less traffic than active monitoring, for example. However, it is more complex compared to the active approach [52].

In an active monitoring paradigm, sensor nodes continuously transmit updated or aware messages to the BS, informing it of their presence and updating it on its status. With an active diagnosis, a series of messages are sent to the BS in order to keep track of the status of the nodes [19,28]. The delay of error diagnosis is reduced in this case; nevertheless, delivering consecutive messages increases the amount of traffic that must be carried by the WSN. Furthermore, transmitting a massive number of messages mains to a rise in the energy usage of sensor nodes, making the approach inefficient in terms of energy usage. In proactive diagnosis, the structure dynamically collects and analyzes data from a network to diagnose previous occurrences and anticipate future events in order to keep the WSN operating at peak performance levels. Compared to other techniques, this error detection method’s accuracy is higher. However, the process of implementing training and testing stages leads to increased latency, which is particularly noticeable in real-time applications of WSNs. The isolation technique is achieved via reactive monitoring, which is the last type of error diagnosis. This method collects status data from the WSN to see if any noteworthy measures have occurred and then takes adaptive steps to reorganize the network [53]. The management system isolates a fault once it has been located. Reactive techniques look for faults by comparing parameters to thresholds or assessing data correlation. Compared to proactive approaches, the methods are less complex but more accurate [30].

### 4.3. Error Recovery

WSNs are restored or reconstructed so that damaged nodes do not hurt the network’s optimum performance; this is the true meaning of the “recovery”. Recovery is defined as the process of replacing a faulty condition with an ideal one [20]. Forward and backward recovery are the two fault recovery techniques that may be used depending on the fault [19].

Backward recovery is used to restore a malfunctioning network to a good condition. This technique requires recording the network’s status at every instant and recovering it. One of the most utilized methods to record the present status of the network is the check-pointing technique [21]. The checkpoint technique saves data and restores them when it changes. This method also retains data, but only changes are recorded. The primary benefit of these techniques is that they are neither network nor process dependent. Their primary issue is that network recovery is costly. There is also no guarantee that the same or comparable problems will not recur in the future. Asset aside in the checkpoint, information is available, and recovery takes place quicker, which is the key benefit of a backward recovery approach. Furthermore, the cost of implementation is cheap; thus, there is no need for redundancy. Nevertheless, it is more difficult to choose an appropriate location to store the network status. Furthermore, the storage of the network state requires huge messaging and thus generates a higher energy usage. In addition, the error cannot be retrieved when the check-point is faulty. If a failure occurs in the network, a set of redundancy devices is placed in the network and is triggered in the event of the failure. In contrast to the previous approach, forward recovery restores the network to a normal condition, allowing it to continue its mission without interruption. Compared to the previous technique, this one is less complicated, and it is unnecessary to know the specific kind of error [19]. When a failure occurs in this network, the network’s state is reset to a new state, increasing the time required for recovery. Additionally, redundancy increases the cost of the network and cannot be incorporated into all sensor neks.

## 5. Proposed Performance Parameters within Fault Tolerance Technique in WSNs

In recent years, various studies have been done to enhance the fault tolerance concept in WSNs. Numerous studies dealt with open issues and challenging matters to reach to ideal fault tolerance structure as referred to in Table 2. Many of these studies used different performance metrics to compare and evaluate different fault-tolerance approaches. Moreover, some researchers are involved to optimize some specific performances during the design stage of fault tolerance structure [54,55]. These different performance metrics represent the main evaluation characteristics of the fault-tolerance approach design. Many of these metrics include detection accuracy, delay, energy consumption, scalability, communication cost, network lifetime, and false alarm rate [56,57,58]. Following is the discussion of the main performance parameters that fault management schemes use in detail.

⬝ Detection Accuracy (DA): The ratio between the successfully recognized faulty sensor nodes divided by the total number of actual defective nodes represents the detection accuracy [19]. Improving error detection accuracy is possible by growing the number of nodes that involved in the fault detection process inside a specific region [59]. Therefore, collaboration among all neighbors in the same event region for example will enhance error detection in general. Increasing fault detection time also increases accuracy even though it will cause greater delay and more energy cost.
⬝ Energy Consumption: Energy consumption is considered one of the main issues in WSNs due to the limited power resource and the complexity or impossibility of replacing the power supplies for all nodes within the WSN [60]. Enhance the energy consumption and network failure control go hand in hand. Therefore, a fault tolerance system is needed to identify and recover problems with low energy usage [58,59]. Reducing the sending operations to the BS will play a vital role to improve energy consumption [61]. Less messaging reduces energy usage in fault control while Increasing fault detection accuracy increases energy usage.
⬝ Delay: Is well-defined as the amount of time that elapses between the occurrence of a fault and the discovery of the error. A longer delay increases the likelihood of a failure spreading inside the network and affects entire network reliability as a consequence of the delay [62].
⬝ Scalability: Many important aspects in WSNs such as fault tolerance and routing, should have the ability to be scalable. Scalability means the network’s capacity to accept more sensor nodes or cluster heads. The fault tolerance approach must be able to manage the high scale and small networks [24].
⬝ Communication Cost: Total number of messages transmitted per node is the communication cost. Because of the significant effect of this activity on the network performance, several fault tolerance approaches have attempted to minimize communication costs to a minimum [59]. However, increased congestion, increased delay, and increased energy usage are all consequences of high communication costs.
⬝ Network Lifetime: A network’s lifespan is defined as the period between network initiation and the moment when the first node dies in the network [63,64]. The fault-tolerance approaches have to take into account the network lifetime and try within its functionality to avoid minimizing the network lifespan.
⬝ False Alarm Rate (FAR): The ratio between the number of faulty nodes that reported error reports to the total number of faulty nodes [59]. In many situations, there are special cases in which some nodes produce an error report towards the BS, especially with monitoring applications. Fault tolerance approaches have a harsh fight with the wrong fault alarms that consume energy, congest the network, and disturb the control center with incorrect readings [65,66]. Such a fake alarm will affect the network’s integrity and reliability.

## 6. Proposed Classification of Fault Tolerance Management Approaches in WSN

**Figure 3:**
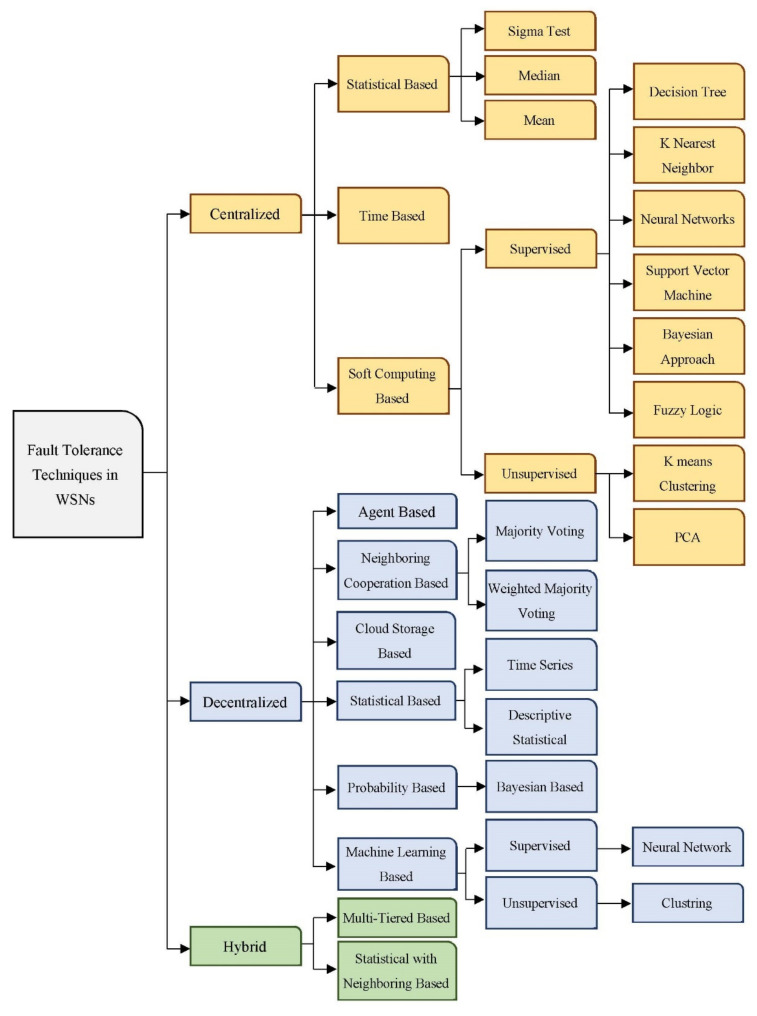
General taxonomy of fault tolerance approaches in WSNs.

Generally, no single fault tolerance structure fits all WSN applications due to its variety and wide use [67]. Many approaches and frameworks have been proposed for the same primary purpose: to satisfy the fault-tolerance concept to gain a high level of reliability and integrity. A general categorization of fault management mechanisms is introduced in this section to make the representation of these schemes more understandable. The suggested categorization divided fault management structures into centralized, decentralized, and hybrid. Each category is subdivided into many subcategories. Figure 3 illustrates the categorization of fault management schemes that have been suggested.

### 6.1. Centralized Fault Tolerance Approaches

The center administrator or BS takes responsibility for fault detection and occurrence choices. By regularly injecting network status queries into the network to collect state information and evaluate this information to find faults, the BS identifies and handles all errors in the WSN. Although this method is easier for smaller networks, it has several drawbacks, including high message traffic near the BS and high energy usage [68]. 

Based on their effectiveness, centralized approaches may be divided into statistical-based, soft computing-based, and time-based. With statistical methods, the statistics are transmitted to the BS and aggregated; then, it is examined to be assessed via the fault tolerance framework [69]. This approach uses statistical methods to identify outliers in the data set under consideration, such as the sigma test, median, and mean.

Methods based on soft computing are algorithms primarily focused on machine learning methods [70]. There are two types of learning methods: supervised learning and unsupervised learning. In supervised learning, an input-output collection is provided to a system, and the system is instructed to train a given input to outcome pairs in the group. To train the system, this technique needs some input data. Neural networks, support vector machines, K-nearest neighbor, Bayesian statistics, decision trees, and fuzzy logic are examples of learning methods [21,24,31,62]. However, in certain situations, supervised learning will not provide the desired results. Another machine learning technique is unsupervised learning. Learning is done on un-marked raw data to uncover unseen forms in unsupervised learning. Principal Component Analysis (PCA) and K-means clustering are examples of unsupervised learning [71].

In time-based fault tolerance approaches, nodes utilize Carrier-Sense Multiple Access with Collision Avoidance (CSMA/CA) and constantly listen to the medium while the network is deployed. To begin, the BS builds a tree structure that links nodes and routes traffic. Data from adjacent nodes is collected at this stage. Finally, the BS allocates a slot to each sensor node for information transmission. Many slots are also allocated to nodes for time synchronization and error handling. Nodes use CSMA/CA for communication listening during the listening time to identify problems [72]. Even though these methods depend on the nodes to detect the errors, the BS will make the main decision. As aforementioned, all centralized approaches suffer from high overhead and lack in scalability matter even though there are simple to implement. Generally, centralized methods have many drawbacks. First, because of the network’s size and density, a lot of information is communicated to the BS, rapidly depleting the energy of nodes nearby. Centralized paradigms are incompatible with large networks. The approaches also need a huge database to hold a huge number of data, increasing installation costs. Additionally, the BS is a weak point in centralized systems and it may have its own errors. When it fails, the output is inaccurate or absent. A faulty BS is tough to replace in many environments. Because the BS receives all network data, it becomes congested, affecting network performance. Lastly, centralized approaches transmit a huge amount of information over the wireless network to obtain information about its status, leading to increased energy consumption, bandwidth waste, and scalability issues [73].

### 6.2. Decentralized Fault Tolerance Approaches

The decentralized fault-tolerant mechanisms will be tackled particularly in this sub-section. Unlike centralized control, these structures use numerous management stations spread throughout the whole wireless network. In decentralized frameworks, each node, cluster head, backbone node, or master node is in charge of a portion of the network. It has the ability to interact directly with other nodes to execute fault detection tasks performed by the BS in the last category [19]. In distributed systems, sensor nodes control their resources and management systems. There is less need to communicate with BS when the nodes can make decisions regarding their status. In terms of functionality, distributed fault-tolerant structures are divided into six categories: neighborhood cooperation-based, statistical-based, probability-based, machine learning-based, cloud storage-based, and agent-based. The basic idea behind the neighborhood-based techniques is a correlation among nodes in the same region [74]. 

Neighborhood voting may be split into majority voting and weighted majority voting. To determine the fault state of nodes, the majority of votes presume that neighboring nodes have the majority of error situations. For each node in the WSN, the weighted majority approach gathers weighted votes from all nearby nodes and forecasts a higher number of votes. Statistical methods are algorithms that identify errors in data using analytical techniques. Time-series-based and descriptive statistical-based are two subcategories of statistical methods. The time-series approach examines time-series data to identify patterns and calculate variations. Deviations in WSNs data are detected using tests. One of the preferable tests is the Kolmorgov Smirnov [75]. On the other hand, descriptive statistical-based techniques are for determining defects that utilize one of the central tendency metrics, such as the mean of neighborhood nodes. Probability fault tolerance methods rely on the probability of node failure to identify the fault state of nodes in a distributed network environment

A node’s fault probability and the fault probability of its neighbors are used to compute the posterior fault probability, which is then used to identify the faulty nodes. Based on the Bayes theorem, Bayesian statistical approaches are used to determine the probability that a node is inaccurate. Machine-learning methods are a subclass of decentralized approaches that have lately received a lot of interest [76]. 

These approaches may be divided into supervised and non-supervised detection techniques. Training data sets are used in supervised error detection methods to learn the difference between real and error data and to anticipate many sensor failures.

The node’s weight is used in neural network-based methods to anticipate data mistakes. Unlike supervised learning methods, unsupervised learning methods have not been given any datasets to work with and have not trained with any database. This area includes clustering methods. Clustering-based methods group nodes into different clusters and link them to a cluster head that examines each node. In agent-based algorithms, the ultimate error status of a sensor node is decided by agents chosen from across the WSN or by the sensor nodes themselves, depending on the methodology. Even though these methods use various information from neighbors, individual nodes or agents make the ultimate choice [77]. Cloud-based methods take advantage of cloud-based resources to decrease the cost of computing tasks [78].

The basic concept behind this method is to move the input data from the nodes to cloud storage and then utilize map reduction to parallelize the error detection process, which would decrease the time it takes to identify faults in the entire system [79]. However, this method is not used commonly in WSN.

The goal of decentralized fault tolerance approaches is to solve the issues that centralized fault management frameworks have, such as increasing energy efficiency and minimizing the total overhead [19,27]. Various numbers of nodes manage faults to achieve the goal instead of entirely depending on BS. However, distributed fault management systems still suffer from delays. They concentrate on lowering energy usage and increasing the accuracy of problem detection. The structures based on neighbor collaboration are focused on improving fault detection accuracy. Neighbor cooperation techniques are gaining popularity due to the requirement for more accurate fault tolerance frameworks in WSNs [58,59].

### 6.3. Hybrid Fault Tolerance Approaches

The last category in the proposed taxonomy is the hybrid fault tolerance structure, a combination of centralized and decentralized management approaches. Hybrid approaches can be divided into two main subcategories: multi-tiered based and statistical with neighboring based [59]. Hybrid algorithms are employed in a large multi WSN, where nodes are grouped into clusters with cluster heads [80]. Each cluster’s nodes transmit their information to the cluster leaders. Cluster heads then send the data to a central base station for processing [2]. In the trust matrix method, a trust matrix is utilized to assess the trustworthiness of data. Hybrid algorithms also combine many detection methods that have been mentioned before into a single algorithm. 

An example of this category is neighborhood algorithms in conjunction with descriptive statistical methods like mean and median. Hybrid methods’ main goal is to reduce energy usage and reduce the delay in fault detection. The fault detection time is minimal since nodes are responsible for detecting their own problems. Furthermore, implementing a fault tolerance system in the cluster heads and master nodes lowers node energy usage since nodes with more energy can detect and recover problems. However, the correct distribution of clusters in a network and their distance from the BS cause the network to become more complicated [81].

## 7. Comparative Analysis, Discussion, and Open Issues 

A total of 62 scientific papers have been synthesized and have been reviewed. The collection of these scientific articles has been selected carefully to cover all the previous fault tolerance structures and techniques. The basic information for every article, the explanation of each methodology, and the representation of all performance metrics have been included in Table 3. The performance metrics for each study have been used to clarify the enhancement and modification of previous works.

Table 4 classified the aforementioned scientific articles according to error types, fault tolerance approaches, and fault management structure detailed in Section 3, Section 4 and Section 6, according to the main parameters related to presenting and designing an efficient fault management structure for WSNs, represented in Section 5. The constraints include detection accuracy, energy consumption, latency, scalability, and communication cost, among others. The assessment of the existing frameworks is shown in Appendix A.

After analyzing various fault management architectures while considering the multiple parameters discussed in Section 5, our survey displayed and synthesized the findings in Table A1 (Appendix A). The review study examined the energy consumption of various fault management methodologies based on the data collected from energy consumption. As fault management systems’ energy usage decreases, nodes have longer lifetimes, resulting in an increase in the total lifespan of the network as a consequence [135]. 

Most centralized fault tolerance techniques exhaust a tremendous amount of power due to the high sending operations toward the BS. Unlike centralized fault tolerance approaches, the majority of decentralized and hybrid fault tolerance approaches minimize energy consumption. In the same context of the speech, the centralized fault management frameworks do not involve the error recovery process in deep, which results in keeping fault data moving back and forth inside the network, causing more and more energy consumption.

A primary strategy for estimating network congestion in fault tolerance techniques is based on the amount of traffic flowing through the network. It is possible to employ this strategy by examining the number of error messages that have been issued and received over time. As a result of using the congestion control strategy, the traffic load for the complete fault management structure is improved [136]. Centralized approaches congest the WSN since all the sensed data (fault measurements and true events) are forwarded toward the BS for central processing [59]. 

On the other hand, decentralized methods keep the traffic flow low, and fewer messages are kept passing among neighboring nodes. Hybrid fault tolerance methods have the second-highest congestion level because they force the central station to be involved in some steps of their procedures. What should be mentioned here, according to the error diagnosis phase, is that any fault tolerance structure used in the active technique also produces high congestion and consumes more energy.

The false alarm rate in various fault management systems has discussed in Table A1 (Appendix A). The false alarm rate examines the number of malfunctioning nodes that reported problems to the base station and the overall number of faulty nodes. When the number of malfunctioning sensor nodes in a single location is large, the rate of false alarms grows considerably [37,59]. Many neighborhoods’ cooperation-based approaches, statistically based methods, and machine learning-based methods have a low false alarm rate compared to other methods. Furthermore, we examined the error recovery techniques that are used to diagnose faults in order to assess the delay of fault tolerance structure since the time that elapses between the incidence of an error and the discovery of the failure is the fundamental idea of delay [137]. Any fault management system that includes an active error diagnosis approach and backward monitoring recovery approach will incur reduced latency as a result of these considerations. Aside from that, employing a mobile sink inside the same network will result in less latency overall. It should go without saying that there is a link between fault detection accuracy and the overall time taken to discover an issue [19]. 

Consequently, boosting the precision of mistake detection will result in a significant increase in latency. The outcome of studying decentralized fault management solutions reveals that these techniques continue to encounter a delay, mainly because most of these approaches focus on reducing energy usage and increasing detection accuracy.

To estimate the cost of a fault tolerance structure, it was necessary to utilize a calculation dependent on the number of nodes in which the error detection and recovery techniques were implemented [21,31]. Therefore, centralized and hybrid techniques are less expensive to adopt as compared to decentralized ones in terms of implementation costs [19]. To estimate the scalability of the fault tolerance approach, this study examines the changing number of nodes in different frameworks because the scalability concept is related to the ability to increase the number of nodes. Therefore, the clustering method, especially decentralized methods, is generally more scalable than other methods. Lastly, the evaluation of network lifespan came to a basic conclusion. The decentralized fault tolerance management frameworks maximize the network lifespan because many procedures within their work prevent the sensor nodes from consuming their power resource rapidly [59].

Some core challenges attracted our attention through analyzing various fault-tolerance approaches. First, neighboring cooperation-based techniques within the decentralized category provided low traffic. Unlike other strategies, these approaches do not depend on the BS in their operations. Second, they have a low false alarm rate compared with many other methods [37,59]. However, neighboring cooperation-based approaches can be enhanced and renovated by optimizing the majority voting techniques and eliminating the source of the faults. More investigation on these open issues could improve the performance of the decentralized approach, especially when embedded with a routing algorithm.

## 8. Open Research Issues

FT term is related directly to network reliability and data integrity. Thus, there is a real need to provide real attention to this concept. Novel techniques must be discovered to build and propose more suitable and satisfactory fault tolerance structures in WSNs. Therefore, overcoming current problems and challenges is crucial. This section summarizes five challenging open issues, and the aim is to provide attractively and still stand research directions for other researchers. In the following, the open research issues are presented according to the proposed taxonomy of current fault tolerance approaches.

### 8.1. Energy Efficiency

Energy efficiency is one of the significant concerns in WSNs. It is essential to consider the energy-efficient related issues incurred by any algorithm due to its respective design for WSNs [16]. In one way or another, all FT techniques consume power to accomplish the fault detection phase. However, there is a difference in the consumed energy amount depending on the different fault-tolerance approaches regarding the main three categories in the fault tolerance structures.

Centralized approaches, for example, waste more energy than other approaches [59], which represents the main issue that still stands with these kinds of strategies. This is due to the massive amount of sensed data that are sent to the BS. Analyzing the sensed data in a centralized way is insufficient and should be organized well.

### 8.2. Communication Overhead 

Overhead still represents a challenging task in fault tolerance management. Numerous studies proposed several approaches to minimize the overhead during error detection, diagnosis, and recovery. However, most fault tolerance algorithms suffer from high overhead at the node level, especially decentralized ones. For instance, the neighboring cooperation is based on exchanging many control messages among neighbors to gain high detection accuracy far from the central BS [138]. Such actions come with a high overhead as the network becomes more crowded.

### 8.3. Security

Security in WSNs is one of the critical requirements. Taking the fault-tolerance concept into consideration, there is a clear correlation between security and faults in the environment of the WSN. Errors will increase the doubt term and make the protection from attackers even more complicated. An intrusion can cause faults. Additionally, faults dramatically can allow and facilitate a new intrusion to attack the WSN [17]. Moreover, disambiguation between a faulty node and a malicious node is a tricky task in WSNs needs to be investigated widely.

### 8.4. Scalability and Density Deployment 

The evaluation process performed on the previous studies clarifies that scalability and density deployment of the nodes are restricted and have high requirements that need to be handled. For example, centralized approaches are not fit for the large-scale networks and do not provide the scalability option to run additional new nodes added to the network. 

Unlike centralized approaches, hybrid and decentralized methods are more appropriate for networks that constantly gain more nodes. However, these approaches’ performance decreases gradually in the high-density deployment of nodes. This is because high-density deployment requires an extra layer of complexity in terms of synchronization and location system of nodes [139].

### 8.5. Latency

Latency time is a high priority in WSNs because faults must be detected and eliminated from the network as soon as possible. In many real-time applications, responses that take a long period of time may pose a high risk in certain situations. Latency represents a continued open issue in the fault-tolerance methods due to the average time taken to detect and dealt with faults.

All fault tolerance algorithms consume time to finish the error detecting stage and the error recovering phase [59]. However, centralized algorithms have low delay as compared to other approaches since they have all data positioned at the central point. In contrast, decentralized systems, especially those bases on neighbor voting, have a high delay.

## 9. Conclusions

As deliberated extensively, FT refers to the network’s ability to deal successfully with faults, and it is crucial for WSNs. Decreasing overall WSN errors is related to the initial implementation of a fault-tolerance approach which leads to the optimal functionality for the network. Due to its importance for satisfying network reliability, numerous scientific studies have been proposed to develop new structures and techniques. 

This work presented a comprehensive survey of fault tolerance strategies in WSNs, consisting of many main stages. First, we classified error types into five general categories with many subcategories. Second, the study discussed the three main principles in fault tolerance structure: error detection, error diagnosis, and error recovery. Third, this study designed a new taxonomy for the current fault tolerance structures. The proposed taxonomy divided the current techniques into three main classes: centralized, decentralized, and hybrid.

Additionally, our extensively enhanced taxonomy has divided each class into many subclasses. The classification process was based on the nature of the fault tolerance system process, the kind of network topology, the type of fault, the kind of diagnosis process, the type of error recovery, and the performance metrics. Moreover, a brief description of the eight main performance metrics used to evaluate the fault-tolerance approaches has been demonstrated. In addition, a deep analysis was conducted on a broad range of studies from 2016 to May 2022 to estimate the weaknesses in the current fault tolerance approaches using the performance evaluation metrics. Lastly, open issues related to the mentioned term have been presented according to our extensive review.

## Figures and Tables

**Table 1:** Surveys on Fault-Tolerant in WSNs.

Survey Article	Fault Tolerance Framework Classification	Error Classification	Comparative Study	Open Issues	Specific to a Particular Branch of the WSN	Frameworks			Related Works in Term of Time
						1–20	20–40	More than 40	
[17]	✓	✓	✓	✓	✓			✓	1992–2020
[18]	×	×	✓	✓	✓	✓			2014–2019
[19]	✓	✓	✓	✓	×		✓		2003–2018
[20]	×	×	×	✓	×	✓			2013–2015
[21]	✓	✓	✓	×	×		✓		2009–2018
[22]	✓	×	×	×	×	✓			2000–2014
[23]	×	×	✓	✓	×	✓			2006–2014
[24]	✓	✓	✓	✓	×			✓	2000–2014
[25]	✓	✓	✓	×	×		✓		2013–2017
[26]	×	×	✓	×	×		✓		2000–2015
[27]	✓	✓	✓	×	×			✓	2005–2017
[28]	✓	×	✓	×	✓		✓		2008–2017
[29]	✓	×	×	✓	×		✓		2002–2005
[30]	×	×	✓	✓	×	✓			2004–2009
[31]	✓	×	✓	×	×	✓			2002–2009
[32]	×	×	✓	×	×	✓			2002–2007
[33]	✓	✓	✓	×	×	✓			2002–2007
[34]	✓	✓	✓	✓	×		✓		2002–2006

**Table 2:** Surveys Classifications based on Year, Citation, and Main Contribution.

Survey Article	Main Contribution
[17]	Presented a comprehensive review of fault-tolerant approaches developed for Underwater Sensor Networks (USNs).
[18]	Presented new future directions and unsolved issues in routing protocols for Flying Ad Hoc Network (VANET). One issue is related to the critical need for having a high fault tolerance ability embedded with routing protocols.
[19]	Presented a summarization and analysis of many previous fault management frameworks developed and designed for WSN.
[20]	Presented a review of the fault-tolerant strategies used to create trustworthy WSNs.
[21]	Presented and analyzed a group of methods for fault detection in WSNs. The study showed a need for a clearer, more accurate, and more comprehensive fault detection and fault tolerance strategy that would maximize the energy savings of the sensor nodes.
[22]	Presented a discussion on previous and fundamental in the context of time of fault tolerance algorithms that deals with errors and radiation effects on sensor behavior.
[23]	Presented a study on different fault recovery techniques and analyzed their methodology in terms of energy use.
[24]	Presented a discussion of some approaches used not just for fault detection but also to prevent faults from occurring, such as data aggregation. The authors classified the fault tolerance approaches according to only two factors: the number of nodes and the region size.
[25]	Presented a classification of fault diagnosis approaches (From 2013 to 2018) into three categories based on the decision hubs and key characteristics of employed algorithms.
[26]	Presented an analysis for specific methods in fault tolerance such as deployment, redundancy, and clustering.
[27]	Presented state of the art for self-healing techniques. The study divided the self-healing mechanisms into four steps: information collection, fault detection, fault classification, and fault recovery.
[28]	Presented a detail review on the sensor nodes failures detection and fault tolerance in Ambient Assisted Living (AAL) systems based on WSNs.
[29]	Presented a brief investigation of many problems that a sensor node may encounter with a general classification of fault tolerance structure.
[30]	Presented a comparative study for several fault management techniques and compared them according to dominant criteria such as overhead, bandwidth, and scalability.
[31]	Presented a comprehensive review of several approaches to the notion of fault tolerance. The study proposed a categorization for fault frameworks based on the structure of task management.
[32]	Presented a summarization of the key ideas for existing fault-tolerant techniques in routing protocols in WSNs.
[33]	Presented a review of frameworks for particular applications and then categorized various fault management according to the types of problems that occur in each implementation.
[34]	Presented a new approach related to the security risks that must be handled throughout all operating stages of a fault-tolerant system in WSN.

**Table 3:** Primary Information, Methodology, and Performance Metrics.

References	Area of Study	Methodology	Main Performance Metrics
[2]	Internet of Things (IoT) and Wireless Sensor Networks (WSNs)	MATLAB	Network Lifetime. Number of Dead Cluster Head. Number of Dead Sensor Node. Average Succuss Rate. Average Survival Rate. Average End to End (E2E) Delay.
[7]	Wireless Sensor Networks (WSNs)	MATLAB	Residual Signal. Weighting Fault Signals. States Responses of the Distributed Fuzzy Filters. Disturbance Input and Fault Input.
[13]	Wireless Sensor Networks (WSNs)	MATLAB	Energy Consumption. E2E Delay. Total Throughput.
[14]	Wireless Sensor Networks (WSNs)	MATLAB	Detection Accuracy.
[37]	Internet of Things (IoT) and Wireless Sensor Networks (WSNs)	NS3	False Positive Rate. Fault Detection Accuracy. False Alarm Rate. Network Lifetime. Throughput.
[45]	Wireless Sensor Networks (WSNs)	MATLAB	Energy Balance. Intrusion Tolerance. Fault tolerance.
[46]	Wireless Sensor Networks (WSNs)	MATLAB	Residing Energy. Energy Consumption. Number of Cluster Heads. Network Lifetime.
[57]	Industry Revolution (IR 4.0) and Internet of Things (IoT)	Statistical Model	Probability of Detection. Probability of False Alarm.
[59]	Wireless Sensor Networks (WSNs)	NS2	Packet Error Rate. Latency. Network Lifespan. False Alarm Rate. Detection Accuracy.
[82]	Wireless Sensor Actor Networks (WSANs)	Castalia	Detection Accuracy. Message Received Per Node. False Alarm Rate. Message Sent Per Node.
[83]	Wireless Sensor Networks (WSNs)	Python	Accuracy. Precision. F1 score/F Measures. Training Time.
[84]	Wireless Sensor Networks (WSNs)	OMNET++	Network Lifetime. Packet Loss Rate. E2E Delay.
[85]	Wireless Sensor Networks (WSNs)	MATLAB	Localization Accuracy. Localizations Errors. Fault Ratio.
[86]	Wireless Sensor Networks (WSNs)	Testbed	E2E. Deployment Cost. Number of Bad Links in each Path.
[87]	Wireless Sensor Networks (WSNs)	Testbed	Fault Response Time. Detection Accuracy. False Alarm Rate.
[88]	Wireless Sensor Networks (WSNs)	Vienna Scientific Cluster VSC	Communication Cost. Average Message per Node. Communication Overhead.
[89]	Wireless Sensor Networks (WSNs)	Castalia	Fault Recovery Time. Consumed Energy. Network Lifetime.
[90]	Underwater Wireless Sensor Networks (UW_WSNs)	NS2	Network Lifetime. Recovery of Nodes. Probability of Failure Nodes. Coverage Ratio.
[91]	Wireless Sensor Networks (WSNs)	MATLAB	Fault Detection Accuracy. False Alarm Rate. Energy Cost. Network Lifetime.
[92]	Wireless Sensor Networks (WSNs)	Testbed and TOSSIM	Energy Consumption. Network Lifetime. Received Byte Account. Transmitted Byte Account.
[93]	Internet of Things (IoT) and Wireless Sensor Networks (WSNs)	NS2	Total Throughput. E2E. Network Lifetime. Power Consumption. Hop Count.
[94]	Wireless Sensor Networks (WSNs)	Testbed	Detection Rate. Distance Covered. Recovery Rate.
[95]	Internet of Things (IoT) and Wireless Sensor Networks (WSNs)	NS2	Average Dissipated Energy. Average Delay. Average Packet Delivery Ratio. Functional Complexity.
[96]	Wireless Sensor Networks (WSNs)	NS2	E2E. Throughput. Packet Delivery Ratio. Latency. Packet Loss Rate. Fault Probability.
[97]	Wireless Sensor Networks (WSNs)	MATLAB	Delay. Average Data Loss. Average correct Data. Energy Consumption.
[98]	Wireless Sensor Networks (WSNs)	Testbed	Mean Square Deviation. Fraction of Disconnectivity. Average Path Length.
[99]	Wireless Sensor Networks (WSNs)	Testbed and MATLAB	False Classification Rate. False Alarm Rate. Fault Detection Accuracy. False Positive Rate.
[100]	Wireless Sensor Networks (WSNs)	MATLAB	Fault Detection Accuracy. Fault Probability Rate. False Alarm Rate. Fault Positive Rate.
[101]	Wireless Body Area Network (WBAN)	MATLAB	Packet Transmission Ratio. Average Delay. Energy Saving.
[102]	Wireless Sensor Networks (WSNs)	MATLAB	Average Localization Error is Studied by Varying the Number of Faulty Nodes.
[103]	Wireless Sensor Networks (WSNs)	NS2	False Positive Ratio. Detection Accuracy. Energy Consumption.
[104]	Wireless Sensor Networks (WSNs)	Testbed and NS2	Fault Detection Accuracy. False Positive Rate. Network Overhead.
[105]	Wireless Sensor Networks (WSNs)	Testbed	Fault Detection Performance. Event Detection Performance.
[106]	Wireless Sensor Networks (WSNs)	MATLAB	False Alarm Rate (FAR). Correct Detection Rate (CDR).
[107]	Wireless Sensor Networks (WSNs)	OMNET++	Consumed Energy. Network Lifespan. Classification Accuracy. False Alarm Rate.
[108]	Wireless Sensor Networks (WSNs)	Testbed	True Positive Rate. False Positive Rate. Detection Accuracy. Precision.
[109]	Wireless Sensor Networks	MATLAB	Fault Detection Accuracy. Energy Consumption. False Alarm Rate.
[110]	Industrial Wireless Sensor Networks (IWSNs)	MATLAB	False Alarm Rate. Detection Accuracy.
[111]	Wireless Sensor Networks (WSNs)	NS2	False Alarm Rate. Fault Detection Accuracy. Energy Consumption. Fault Detection Latency. False Positive Rate.
[112]	Wireless Sensor Networks (WSNs)	MATLAB	Remaining Energy. Packet Delivery Ratio. Error Detection Accuracy.
[113]	Wireless Sensor Networks (WSNs)	MATLAB	Sensor Fault Probability. Total Energy Consumption. Detection Accuracy.
[114]	Wireless Sensor Networks (WSNs)	Testbed	Fault Detection Accuracy. Average Error rate. Standard Deviation.
[115]	Wireless Sensor Networks (WSNs)	Testbed and MATLAB	Network Lifetime. Energy consumption. False Alarm Rate. Fault Detection Accuracy.
[116]	Wireless Sensor Networks (WSNs)	NS2	Detection Accuracy. False Alarm Rate. False Positive Rate
[117]	Wireless Sensor Networks (WSNs)	MATLAB	Energy Consumption. Delay. Packet Drop Rate. Delivery Ratio.
[118]	Wireless Sensor Networks (WSNs)	NS2	Detection Time. Percentage of Failure Detection. Mean Detection Time. Percentage of Suspicious. Mean Time to Detect Failure in CHs.
[119]	Mobile Wireless Sensor Networks (WSNs)	OMNET++	Energy Consumption. Packet Drop Rate. Packet Delivery Ratio.
[120]	Wireless Sensor Networks (WSNs)	NS2	Average Delay. Packet Delivery Ratio. Throughput.
[121]	Wireless Sensor Networks (WSNs)	MATLAB	Detection Accuracy Rate. Relative Restoration Error. Energy Consumption Rate.
[122]	Wireless Sensor Networks (WSNs)	Python	Detection Accuracy. Matthews Correlation Coefficient (MCC). True Positive Rate. F1 Score.
[123]	Wireless Sensor Networks (WSNs)	MATLAB	Network Efficiency. Overload-Tolerance Coefficient. Congestion-Tolerance Coefficient. Traffic Variance.
[124]	Wireless Sensor Networks (WSNs)	Simulation	Cooperative Detection Probability. Surveillance Quality.
[125]	Internet of Things (IoT) and Wireless Sensor Networks (WSNs)	C++	Network Energy Consumption. Failure Rate. Deadline Missing Ratio. Network Lifetime.
[126]	Wireless Sensor Networks (WSNs)	Monte Carlo and MATLAB	Probability of a Node Failing. Root Mean Square Error (RMSE). Cumulative Distribution Function (CDF).
[127]	Internet of Things (IoT)	Castalia	Delivery Ratio. E2E Delay. Energy Consumption.
[128]	Wireless Sensor Networks (WSNs)	MATLAB	Detection Accuracy. False Positive Rate.
[129]	Internet of Things (IoT) and Wireless Sensor Networks (WSNs)	NS2	Communication Delay. Fault Tolerance Optimization.
[130]	Internet of Things (IoT) and Wireless Sensor Networks (WSNs)	MATLAB	Throughput. Energy Consumption. Average Delay.
[131]	Internet of Things (IoT) and Wireless Sensor Networks (WSNs)	NS2	Barrier Construction Efficiency. Reliability Index (RI). Energy Cost. Percentage Coverage Area with Time. Percentage of coverage holes.
[132]	Internet of Things (IoT) and Wireless Sensor Networks (WSNs)	NS3	Packet Loss Rate. Throughput. Total Energy Consumption. Latency of Recovery. Number of Dead Nodes.
[133]	5G, Industrial Internet of Things (IIoT) and Wireless Sensor Networks (WSNs)	Python	System Cost. Energy Consumption. Total Delay.
[134]	Internet of Things (IoT) and Wireless Sensor Networks (WSNs)	MATLAB	Network Connectivity. Coverage Efficiency. Hole Recovery.

**Table 4:** Network Type, Fault Type, and Fault Management Structure.

References	Network Type	Fault Type	Fault Tolerance Approach	Fault Tolerance Procedures		
				Detection	Diagnosis	Recovery
[2]	Heterogeneous	Node Faults (CH Failure)	Hybrid Based	Decentralized	Reactive	-
[7]	Homogeneous	Node Faults	Centralized Based	Self-Supervision	Active	-
[13]	Homogeneous	Node Faults and Network Faults	Decentralized Based	Self-Supervision and Decentralized	Proactive	Forward
[14]	Homogeneous	Node Faults	Decentralized Based	Self-Supervision	Reactive	-
[37]	Heterogeneous	Node Faults (CH Faults)	Decentralized Based	Decentralized	Active-Proactive	-
[45]	Heterogeneous	Node Faults (CH Failure)	Decentralized Based	Decentralized	Active	-
[46]	Heterogeneous	Node Faults (CH Faults) and Network Faults (Links)	Decentralized Based	Decentralized	Active	Backward
[57]	Homogeneous	Node Faults	Centralized Based	Centralized	Passive	-
[59]	Homogeneous	Node Faults and Network Faults	Decentralized Based	Decentralized	Reactive	-
[82]	Heterogeneous	Node Faults	Hybrid Based	Decentralized	Active	Backward
[83]	Homogeneous	Node Faults	Centralized Based	Decentralized	Proactive	-
[84]	Heterogeneous	Node Faults	Decentralized Based	Self-Supervision	Active	-
[85]	Heterogeneous	Node Faults	Decentralized Based	Decentralized	Reactive	Forward
[86]	Homogeneous	Node Faults and Network Faults	Centralized Based	Decentralized	Active- Proactive	-
[87]	Homogeneous	Node Faults	Decentralized Based	Decentralized	Passive	-
[88]	Homogeneous	Node Faults	Decentralized Based	Decentralized	Reactive	-
[89]	Heterogeneous	Node Faults	Decentralized Based	Decentralized	Reactive	-
[90]	Heterogeneous	Node Faults	Centralized Based	Self-Supervision	Active	Backward
[91]	Heterogeneous	Node Faults	Centralized Based	Decentralized	Active	Backward
[92]	Heterogeneous	Node Faults	Decentralized Based	Decentralized	Proactive	
[93]	Heterogeneous	Network Faults (Link Failure)	Decentralized Based	Decentralized	Reactive	-
[94]	Homogeneous	Network Faults (Link Failure)	Centralized Based	Centralized	Passive	-
[95]	Heterogeneous	Node Faults	Decentralized Based	Decentralized	Active	Forward
[96]	Homogeneous	Node Faults and Network Faults	Decentralized Based	Decentralized	Active	-
[97]	Heterogeneous	Node Faults and Network Faults	Decentralized Based	Decentralized	Active	Backward
[98]	Heterogeneous	Network Faults (Link Failure)	Decentralized Based	Decentralized	Active	-
[99]	Heterogeneous	Network Faults (Link Failure)	Decentralized Based	Decentralized	Active	-
[100]	Heterogeneous	Node Faults	Decentralized Based	Decentralized	Proactive	-
[101]	Homogeneous	Network Faults (Link Failure)	Centralized Based	Centralized	Passive	-
[102]	Heterogeneous	Node Faults	Decentralized Based	Decentralized	Active	-
[103]	Heterogeneous	Node Faults	Centralized Based	Decentralized	Reactive	-
[104]	Heterogeneous	Node Faults	Decentralized Based	Decentralized	Active	Forward
[105]	Homogeneous	Node Faults	Centralized Based	Centralized	Active and Proactive	-
[106]	Homogeneous	Node Faults	Centralized Based	Self-Supervision and Centralized	Passive	Backward
[107]	Heterogeneous	Node Faults (CH Failure)	Decentralized Based	Self-Supervision and Decentralized	Active	Forward
[108]	Homogeneous	Node Faults	Centralized Based	Centralized	Active	-
[109]	Homogeneous	Node Faults and Network Faults	Centralized Based	Self-Supervision	Active	-
[110]	Heterogeneous	Node Faults	Decentralized Based	Decentralized	Active	Backward
[111]	Heterogeneous	Node Faults	Decentralized Based	Decentralized	Active	-
[112]	Heterogeneous	Node Faults	Hybrid Based	Decentralized	Proactive	-
[113]	Heterogeneous	Node Faults	Decentralized Based	Decentralized	Passive and Active	-
[114]	Homogeneous	Node Faults	Centralized Based	Centralized	Active	-
[115]	Homogeneous	Node Faults	Decentralized Based	Centralized	Active	-
[116]	Heterogeneous	Node Faults	Centralized Based	Decentralized	Passive	Forward
[117]	Heterogeneous	Node Faults	Decentralized Based	Decentralized	Active	Backward
[118]	Heterogeneous	Node Faults (CH Failure)	Centralized Based	Decentralized	Passive and Active	-
[119]	Heterogeneous	Node Faults (CH Failure)	Centralized Based	Centralized	Active	Forward
[120]	Heterogeneous	Node Faults	Decentralized Based	Decentralized	Proactive	Backward
[121]	Heterogeneous	Node Faults	Hybrid Based	Decentralized	Proactive	Backward
[122]	Homogeneous	Node Faults	Decentralized Based	Self-Supervision and Decentralized	Proactive	-
[123]	Heterogeneous	Node Faults and Network Faults	Decentralized Based	Decentralized	Active	-
[124]	Homogeneous	Node Faults	Decentralized Based	Decentralized	Active	-
[125]	Heterogeneous	Node Faults	Decentralized Based	Self-Supervision and Decentralized	Passive	-
[126]	Heterogeneous	Node Faults and Network Faults	Decentralized Based	Self-Supervision	Proactive	-
[127]	Homogeneous	Node Faults	Centralized Based	Centralized	Active	-
[128]	Homogeneous	Node Faults	Centralized Based	Self-Supervision and Centralized	Active	-
[129]	Heterogeneous	Network Faults	Centralized Based	Decentralized	Reactive	-
[130]	Heterogeneous	Node Faults and Network Faults	Decentralized Based	Self-Supervision and Decentralized	Active	-
[131]	Homogeneous	Node Faults and Network Faults	Centralized Based	Self-Supervision	Active	Forward
[132]	Heterogeneous	Node Faults (CH Failure)	Decentralized Based	Decentralized	Active	Backward
[133]	Heterogeneous	Network Faults	Decentralized Based	Decentralized	Active	-
[134]	Heterogeneous	Network Faults	Decentralized Based	Self-Supervision and Decentralized	Active	Forward

## Data Availability

The data that support the findings of this study are available from the corresponding author [G.H.A] upon reasonable request.

## Appendix A

**Table A1:** Analysis and Classification of Fault Tolerance Management Structures.

Techniques	Contributions	Parameters Enhancement within Technique							
		Minimize Energy Use	Minimize Congestion	Minimize False Alarm Rate	Minimize Delay	Fault Detection Accuracy	Improved Costs	High Scalability	Maximize Network Lifespan
[2]	Proposed a novel fault tolerance routing algorithm using a hybrid meta-heuristic algorithm which integrated the Firefly Optimization (FA) with Gray Wolf Optimization (GWO).	✓				✓		✓	✓
[7]	Proposed a novel method for detecting the random packet loss based on the Bernoulli distribution through the network from the sensors to the filters. The proposed method utilizes the IT2 T–S fuzzy model and a new distributed fault detection filter corresponding to the sensor nodes.			✓		✓			✓
[13]	Proposed a new approach based on artificial intelligence to handle the faults during data transmission to the BS.	✓			✓		✓		
[14]	Proposed a novel Distributed Fault Detection (DFD) that recognizes the neighboring hot nodes and imposed their impact for fault detection.		✓			✓	✓		
[37]	Proposed multiple solutions such as a Maximum Coverage Location Problem (MCLP) algorithm to find optimal locations for CH placement, a Multi-Objective Deep Reinforcement Learning (MODRL) for fault detection and fault-free optimal data routing path selection, and presented a mobile sink-based data gathering scheme for better reliability.	✓		✓		✓			✓
[45]	Proposed construction of a regular hexagonal-based clustering scheme (RHCS) of sensor networks and analyzed the reliability of RHCS based on the Markov model. Moreover, this work proposed a scale-free topology evolution mechanism.	✓	✓					✓	✓
[46]	Proposed a management framework that is qualified to provide network fault tolerance that detects and recovers mechanisms for various faults including network nodes and communications between them. The whole work was built on the idea of Check Point Node (CHN) and storing all data temporally.	✓			✓			✓	✓
[57]	Proposed a novel machine-learning-based architecture for detecting anomalies readings from sensors, identifying the faulty ones, and adapting them with suitable estimated data.	✓		✓		✓			
[59]	Proposed the True Event-Driven and Fault Tolerance Routing (TED-FTR) approach for real-time applications in WSNs.	✓		✓		✓	✓		✓
[82]	Proposed the Triple Modular Redundancy (TMR) to monitor radiation levels near and within a nuclear reactor.	✓		✓		✓			✓
[83]	Proposed the Extra Trees Based (ETB) to detect and diagnose different types of faults in an ideal time for WSNs.		✓		✓	✓	✓		
[84]	Proposed the Energy Efficient cluster-based Fault-Tolerant Routing Protocol (EE-FT) that avoids node faults before they occur.	✓			✓	✓		✓	✓
[85]	Proposed fault filtering approach to detect and filter out faulty nodes, making the localization process more fault tolerant.	✓						✓	
[86]	Proposed a K-Set Converging Algorithm (KSCA) to build fault tolerance that can deal with Delay Constrained Relay Node Placement.				✓	✓			
[87]	Proposed Trend Correlation-based Fault detection (TCFD) strategy to detect faulty nodes in WSNs.			✓		✓	✓		
[88]	Proposed a push-flow algorithm for fault tolerance and employing the self-correcting properties of repeated improvement.					✓	✓		
[89]	Presented a comparison among three fault-tolerant routing protocols Multilevel, HDMRP, and EAQHSeN.	✓			✓				✓
[90]	Proposed an error guess, detection, and recovery algorithm using the Markov Chain Monte Carlo procedure for Underwater Wireless Sensor Networks (UW-WSN).					✓			✓
[91]	Proposed Reliable Neuro-Fuzzy Optimization Model (RANDOM) for intra-cluster and inter-cluster fault detection.	✓		✓		✓		✓	✓
[92]	Proposed a distributed fault-tolerant algorithm that deals with a finite number of transient errors based on Connected Dominating Set (CDS).	✓						✓	✓
[93]	Proposed fault-tolerant routing algorithm using Fractional Gaussian Firefly Algorithm (FGFA) and Darwinian Chicken Swarm Optimization (DCSO).	✓			✓		✓		✓
[94]	Proposed Directional NN algorithm directed to the next nearest node (NNNN) reduces data acquisition time while maintaining fault tolerance for links failures.				✓				
[95]	Proposed a path graph flow and Marchenko Pastur distribution for fault detection in cluster heads and normal nodes.	✓			✓	✓	✓	✓	✓
[96]	Proposed node faulty detection method to gain reliable communication in a wireless environment with a lot of obstacles.	✓				✓			✓
[97]	Proposed a fault tolerance technique to detect and diagnose faults, the backup nodes used to recover from faults.	✓				✓		✓	✓
[98]	Proposed a novel approach of decentralized detection over a Small World WSNs to utilize traffic flow between node pairs and result in a robust and low-complexity development.		✓				✓		
[99]	Presented a technique that is capable of diagnosing composite faults on sensor nodes and connections, including hard permanent, soft permanent, intermittent, and transient faults.		✓	✓				✓	
[100]	Proposed an optimized Sup-port Vector Machine (SVM) for fault diagnosis in WSN based on the Gray Wolf Optimization (GWO) classifier that used to detect faults in sensor nodes			✓		✓			
[101]	Proposed energy-efficient fault-tolerance approach to enhance the reliability in the WBAN based on the cooperative communication and net-work coding strategy.	✓				✓			
[102]	Proposed a fault-tolerant approach named clustering-based DV Hop using K means clustering and majority voting methods.					✓		✓	
[103]	Proposed a new technique named Low Energy Fault Detection (LED) to utilize the sequence of data acquired by the sensor to detect certain types of faults.	✓		✓		✓			✓
[104]	Proposed a Fault detection method based on the Gaussian transformation algorithm to detect faulty nodes.			✓		✓			
[105]	Proposed and evaluated the trouble of detecting different kinds of fault data and the guidance of each type on event detection results.	✓				✓			
[106]	Proposed the two-stage error detection algorithms based on spatial-temporal cooperation performed by the BS in WSNs.			✓		✓			
[107]	Proposed a logical Cluster Head system in which the CH, like other nodes in the network, is prone to mistakes. The LEACH procedure has been updated to include intelligent dynamic CH selection based on residual energy and sensor inputs after each round.	✓		✓		✓	✓		✓
[108]	Proposed a comparative study for noise, short-term, and fixed faults caused by low battery and calibration. The study was based on the performance of three popular algorithms which are: Support Vector Machine (SVM), Naive Bayes, and Gradient Lifting Decision Tree (GBDT).	✓		✓		✓	✓		
[109]	Presented the hardware error diagnosis methods that detect the heterogeneous hardware errors such as unit, transmitter, and microcontroller.			✓		✓			
[110]	Proposed an error detection approach for Industrial Wireless Sensor Networks (IWSNs) based on software-defined networks (SDNs).			✓		✓	✓		
[111]	Presented a heterogeneity fault diagnosis protocol via three steps to detect many kinds of errors such as hard, soft, and intermittent.	✓		✓	✓	✓		✓	✓
[112]	Presented a novel approach based on distributed detection and fuzzy logic to detect errors, isolate faulty nodes, and reuse some faulty nodes as relay nodes.					✓		✓	
[113]	Proposed fault detection method based on clustering to achieve high detection process run by CHs without bothering the BS.	✓				✓	✓		
[114]	Proposed a high error detection approach based on double machine learning techniques, which are the neural networks and the Support Vector Machine (SVM).	✓				✓			
[115]	Proposed a novel distributed mobile sink-based fault diagnosis scheme for WSNs by using single hop communication.		✓	✓		✓		✓	
[116]	Proposed a fuzzy multilayer with particle swarm optimization for fault detection in WSNs such as hard, soft, intermittent, and intermittent errors.			✓		✓			
[117]	Proposed a clustering-based method for fault tolerance using the genetic algorithm.	✓			✓				
[118]	Propose a new failure detection methodology for clustered WSNs named Efficient and Accurate Failure Detector (EAFD), which uses two degrees of suspicion to decide if a node has failed.		✓				✓		
[119]	Proposed a cluster-based fault detection and recovery method. False data detection is performed by estimating the accuracy value of each sensor node and then detecting and eliminating outliers.					✓			
[120]	Presented a method for preventing node failures by using the Ad hoc On-Demand Distance Vector (AODV) routing protocol and chick point recovery.	✓				✓			
[121]	Proposed a technique based on the Principal Component Analysis (PCA) to deal with information errors and redundant issues.	✓				✓			
[122]	Proposed comparative analysis for fault detection problem. The study evaluates six methods: Support Vector Machine (SVM), Convolutional Neural Network (CNN), Stochastic Gradient Descent (SGD), Multilayer Perceptron (MLP), Random Forest (RF), and Probabilistic Neural Network (PNN).			✓		✓	✓		
[123]	Proposed a practical cascading standard for WSNs, in which the load function is defined on each node according to a new directional traffic metric. The failed node can recover through a reboot after a specific time delay rather than being forever removed from the network.		✓		✓				✓
[124]	Presented a barrier coverage algorithm, namely Maximizing Cooperative Detection Probability (MCDP), which applies the Probability Sensing Model (PSM) and aims to perpetuate the life of solar-powered WSNs while maximizing the surveillance quality of the constructed barrier. The proposed method is based on calculating the detection probability of each sensor to each grid.	✓							✓
[125]	Proposed a novel optimized fault-tolerant task allocation algorithm for IoT-WSNs called Discrete Particle Swarm Optimization (DPSO). The proposed algorithm employs a frame replication and elimination approach to transmit flow replicas over redundant routes and schedules the flow in time slots to avoid data corruption or the effect on the throughput.	✓		✓		✓			✓
[126]	Proposed a robust localization based on the Received Signal Strength Difference (RSSD) with unknown transmit power and Gaussian mixture noise in the presence of faulty nodes. A Robust Fault-Tolerant Localization (RFLT) technique is proposed also using a Generalized Trust-Region Subproblem (GTRS) framework.		✓				✓		✓
[127]	Presented a replicated gateway structure augmented with energy-efficient real-time Byzantine-resilient data communication protocols. The proposed method enhanced the geographic routing protocol capability of delivering messages in an energy-efficient, even in the presence of voids caused by faulty and malicious sensor nodes.	✓				✓			✓
[128]	Proposed a new classification approach for fault detection in WSNs. The proposed technique is based Support Vector Machines (SVMs) classification method SVM technique can detect many types of faults.			✓		✓			
[129]	Proposed a method for FT in virtualization in WSNs, focusing on heterogeneous networks for service-oriented IoT applications. The proposed approach used an Adapted Nondominated Sorting-based Genetic Algorithm (A-NSGA) to solve the optimization problem within network links.				✓	✓		✓	
[130]	Proposed a bio-inspired Particle Multi-Swarm Optimization (PMSO) routing algorithm to create, recover, and elect k-disjoint paths that tolerate the failure while satisfying the quality-of-service parameters. The proposed work utilizes the use of Cumulative Distribution Function (CDF) for the sensors with an exponentially distributed failure rate.	✓			✓				
[131]	Proposed a fault-tolerant barrier scheduling scheme that satisfies the Quality-of-Service (QoS) requirements of surveillance applications in the presence of faults. The proposed method is based on a novel fully weighted dynamic graph model that can detect and recover faults.	✓					✓		
[132]	Proposed a fault-tolerance approach that combines Static Backup and Dynamic Timing Monitoring (SBDTM) for cluster heads to achieve reliable data acquisition and ensure the reliability of an IoT monitoring system. The proposed method used the Markov model-based cluster head to achieve the reliability of the model.	✓		✓				✓	✓
[133]	Proposed a practical Edge-Intelligent Service Placement Algorithm (EISPA) with the use of Particle Swarm Optimization (PSO).to solve a service continuity problem. The work dealt efficiently with the basic fact that some 5G-and-beyond IIoT applications roam around different regions of the MEC servers.	✓		✓	✓		✓		✓
[134]	Proposed a solution for the connectivity and robustness in IoT networks during disaster recovery actions using a mobile robot. The proposed method is based on the use of the Optimal Localizable K-Coverage (OLKC) strategies to help in hole recovery. Moreover, the developed work presented two optimality requirements to achieve maximum coverage by the proposed OLKC in an unfamiliar, hostile, or harsh environment using the lowest number of nodes.	✓			✓			✓	✓

## References

[1] Nagy J., Oláh J., Erdei E., Máté D., Popp J. (2018). The Role and Impact of Industry 4.0 and the Internet of Things on the Business Strategy of the Value Chain—The Case of Hungary. Sustainability.

[2] Jaiswal K., Anand V. (2022). FAGWO-H: A hybrid method towards fault-tolerant cluster-based routing in wireless sensor network for IoT applications. J. Supercomput..

[3] Dowlatshahi M.B., Rafsanjani M.K., Gupta B.B. (2021). An energy aware grouping memetic algorithm to schedule the sensing activity in WSNs-based IoT for smart cities. Appl. Soft Comput..

[4] Abdali T.-A.N., Hassan R., Aman A.M., Nguyen Q., Al-Khaleefa A. (2021). Hyper-Angle Exploitative Searching for Enabling Multi-Objective Optimization of Fog Computing. Sensors.

[5] Idrees A.K., Al-Qurabat A.K.M. (2021). Energy-Efficient Data Transmission and Aggregation Protocol in Periodic Sensor Networks Based Fog Computing. J. Netw. Syst. Manag..

[6] Abdali T.-A.N., Hassan R., Aman A.H.M., Nguyen Q.N. (2021). Fog Computing Advancement: Concept, Architecture, Applications, Advantages, and Open Issues. IEEE Access.

[7] Gao Y., Xiao F., Liu J., Wang R. (2019). Distributed Soft Fault Detection for Interval Type-2 Fuzzy-Model-Based Stochastic Systems With Wireless Sensor Networks. IEEE Trans. Ind. Inform..

[8] Li L., Dai H., Chen G., Zheng J., Dou W., Wu X. (2019). Radiation Constrained Fair Charging for Wireless Power Transfer. ACM Trans. Sens. Netw..

[9] Hayat H., Griffiths T., Brennan D., Lewis R.P., Barclay M., Weirman C., Philip B., Searle J.R. (2019). The State-of-the-Art of Sensors and Environmental Monitoring Technologies in Buildings. Sensors.

[10] Abdali T.-A., Hassan R., Muniyandi R., Aman A.M., Nguyen Q., Al-Khaleefa A. (2020). Optimized Particle Swarm Optimization Algorithm for the Realization of an Enhanced Energy-Aware Location-Aided Routing Protocol in MANET. Information.

[11] Abdulrab H., Hussin F.A., Aziz A.A., Awang A., Ismail I., Devan P.A.M. (2022). Reliable Fault Tolerant-Based Multipath Routing Model for Industrial Wireless Control Systems. Appl. Sci..

[12] Biradar M., Mathapathi B. Secure, Reliable and Energy Efficient Routing in WSN: A Systematic Literature Survey. Proceedings of the 2021 International Conference on Advances in Electrical, Computing, Communication and Sustainable Technologies (ICAECT).

[13] Menaria V.K., Jain S.C., Nagaraju A., Kumari R., Nayyar A., Hosain E. (2020). NLFFT: A Novel Fault Tolerance Model Using Artificial Intelligence to Improve Performance in Wireless Sensor Networks. IEEE Access.

[14] Gharamaleki M.M., Babaie S. (2020). A New Distributed Fault Detection Method for Wireless Sensor Networks. IEEE Syst. J..

[15] Boukerche A., Pazzi R.W.N., Araujo R.B. (2006). Fault-tolerant wireless sensor network routing protocols for the supervision of context-aware physical environments. J. Parallel Distrib. Comput..

[16] Zagrouba R., Kardi A. (2021). Comparative Study of Energy Efficient Routing Techniques in Wireless Sensor Networks. Information.

[17] Vihman L., Kruusmaa M., Raik J. (2021). Systematic Review of Fault Tolerant Techniques in Underwater Sensor Networks. Sensors.

[18] Sang Q., Wu H., Xing L., Xie P. (2020). Review and Comparison of Emerging Routing Protocols in Flying Ad Hoc Networks. Symmetry.

[19] Moridi E., Haghparast M., Hosseinzadeh M., Jassbi S.J. (2020). Fault management frameworks in wireless sensor networks: A survey. Comput. Commun..

[20] Shyama M., Pillai A.S. Fault Tolerance strategies for Wireless Sensor Networks—A Comprehensive Survey. Proceedings of the 2018 3rd International Conference on Inventive Computation Technologies (ICICT).

[21] Effah E., Thiare O. (2018). Survey: Faults, Fault Detection and Fault Tolerance Techniques in Wireless Sensor Networks. Int. J. Comput. Sci. Inf. Secure.

[22] Dhanoriya S., Pandey M. A survey on wireless sensor networks: Faults, misbehaviour and protection against them. Proceedings of the 2017 8th International Conference on Computing, Communication and Networking Technologies (ICCCNT).

[23] Mitra S., Das A., Mazumder S. Comparative study of fault recovery techniques in Wireless Sensor Network. Proceedings of the 2016 IEEE International WIE Conference on Electrical and Computer Engineering (WIECON-ECE).

[24] Chouikhi S., El Korbi I., Ghamri-Doudane Y., Saidane L.A. (2015). A survey on fault tolerance in small and large scale wireless sensor networks. Comput. Commun..

[25] Zhang Z., Mehmood A., Shu L., Huo Z., Zhang Y., Mukherjee M. (2018). A Survey on Fault Diagnosis in Wireless Sensor Networks. IEEE Access.

[26] Kakamanshadi G., Gupta S., Singh S. A survey on fault tolerance techniques in Wireless Sensor Networks. Proceedings of the 2015 International Conference on Green Computing and Internet of Things, ICGCIoT 2015.

[27] Diaz S., Mendez D., Kraemer R. (2019). A Review on Self-Healing and Self-Organizing Techniques for Wireless Sensor Networks. J. Circuits Syst. Comput..

[28] ElHady N.E., Provost J. (2018). A Systematic Survey on Sensor Failure Detection and Fault-Tolerance in Ambient Assisted Living. Sensors.

[29] Kshirsagar R.V., Jirapure A.B. A Survey on Fault Detection and Fault Tolerance in Wireless Sensor Networks. Proceedings of the International Conference on Benchmarks in Engineering Science and Technology ICBEST.

[30] Mitra S., De Sarkar A., Roy S. A review of fault management system in wireless sensor network. Proceedings of the CUBE International Information Technology Conference on-CUBE’12.

[31] Huangshui H., Guihe Q. Fault Management Frameworks in Wireless Sensor Networks. Proceedings of the 2011 Fourth International Conference on Intelligent Computation Technology and Automation.

[32] Alwan H., Agarwal A. A Survey on Fault Tolerant Routing Techniques in Wireless Sensor Networks. Proceedings of the 2009 Third International Conference on Sensor Technologies and Applications.

[33] Khan M.Z., Merabti M., Askwith B. Design Considerations for Fault Management in Wireless Sensor Networks. Proceedings of the 10th Annual PostGradute Symposium on the Conference of Convergence of Telecommunications, Networking and Broadcasting, PGNet 2009.

[34] Kakamanshadi G., Gupta S., Singh S. (2007). A survey on fault tolerance techniques in Wireless Sensor Networks. Interner Bericht. Fakultät für Informatik.

[35] Jiang P. (2009). A New Method for Node Fault Detection in Wireless Sensor Networks. Sensors.

[36] Alansari Z., Prasanth A., Belgaum M.R. A Comparison Analysis of Fault Detection Algorithms in Wireless Sensor Networks. Proceedings of the 2018 International Conference on Innovation and Intelligence for Informatics, Computing and Technologies (3ICT).

[37] Agarwal V., Tapaswi S., Chanak P. (2022). Intelligent Fault-Tolerance Data Routing Scheme for IoT-enabled WSNs. IEEE Int. Things J..

[38] Krivulya G., Skarga-Bandurova I., Tatarchenko Z., Seredina O., Shcherbakova M., Shcherbakov E. An Intelligent Functional Diagnostics of Wireless Sensor Network. Proceedings of the 2019 7th International Conference on Future Internet of Things and Cloud Workshops (FiCloudW).

[39] Saeed U., Lee Y.-D., Jan S., Koo I. (2021). CAFD: Context-Aware Fault Diagnostic Scheme towards Sensor Faults Utilizing Machine Learning. Sensors.

[40] Moridi E., Haghparast M., Hosseinzadeh M., Jassbi S.J. (2020). Novel Fault Management Framework Using Markov Chain in Wireless Sensor Networks: FMMC. Wirel. Pers. Commun..

[41] Boussif A., Ghazel M., Basilio J.C. (2021). Intermittent fault diagnosability of discrete event systems: An overview of automaton-based approaches. Discret. Event Dyn. Syst..

[42] Chen L., Li G., Huang G. (2021). A hypergrid based adaptive learning method for detecting data faults in wireless sensor networks. Inf. Sci..

[43] Swain R.R., Khilar P.M., Bhoi S.K. (2020). Underlying and Persistence Fault Diagnosis in Wireless Sensor Networks Using Majority Neighbors Co-ordination Approach. Wirel. Pers. Commun..

[44] Salayma M., Al-Dubai A., Romdhani I., Nasser Y. (2017). Wireless Body Area Network (WBAN). ACM Comput. Surv..

[45] Hu S., Li G. (2018). Fault-Tolerant Clustering Topology Evolution Mechanism of Wireless Sensor Networks. IEEE Access.

[46] Cheraghlou M.N., Khadem-Zadeh A., Haghparast M. (2017). Increasing Lifetime and Fault Tolerance Capability in Wireless Sensor Networks by Providing a Novel Management Framework. Wirel. Pers. Commun..

[47] Silva I., Guedes L.A., Portugal P., Vasques F. (2012). Reliability and Availability Evaluation of Wireless Sensor Networks for Industrial Applications. Sensors.

[48] Krishnamachari B., Iyengar S. (2004). Distributed Bayesian algorithms for fault-tolerant event region detection in wireless sensor networks. IEEE Trans. Comput..

[49] Lau B.C., Ma E.W., Chow T.W. (2014). Probabilistic fault detector for Wireless Sensor Network. Expert Syst. Appl..

[50] Chen J., Kher S., Somani A. Distributed fault detection of wireless sensor networks. Proceedings of the 2006 Workshop on Dependability Issues in Wireless ad Hoc Networks and Sensor Networks-DIWANS’06.

[51] Mohapatra S., Khilar P.M. (2020). Fault diagnosis in wireless sensor network using negative selection algorithm and support vector machine. Comput. Intell..

[52] Liu K., Li M., Yang X., Jiang M. (2010). Passive diagnosis for wireless sensor networks. IEEE/ACM Trans. Netw..

[53] Butun I., Morgera S.D., Sankar R. (2014). A Survey of Intrusion Detection Systems in Wireless Sensor Networks. IEEE Commun. Surv. Tutor..

[54] Paradis L., Han Q. (2007). A Survey of Fault Management in Wireless Sensor Networks. J. Netw. Syst. Manag..

[55] Chou C.T., Ignjatovic A., Hu W. (2013). Efficient Computation of Robust Average of Compressive Sensing Data in Wireless Sensor Networks in the Presence of Sensor Faults. IEEE Trans. Parallel Distrib. Syst..

[56] Darvishi H., Ciuonzo D., Eide E.R., Rossi P.S. (2021). Sensor-Fault Detection, Isolation and Accommodation for Digital Twins via Modular Data-Driven Architecture. IEEE Sens. J..

[57] Shahraki A., Taherkordi A., Haugen O., Eliassen F. (2021). A Survey and Future Directions on Clustering: From WSNs to IoT and Modern Networking Paradigms. IEEE Trans. Netw. Serv. Manag..

[58] Seyfollahi A., Ghaffari A. (2020). A lightweight load balancing and route minimizing solution for routing protocol for low-power and lossy networks. Comput. Netw..

[59] Biswas P., Samanta T. (2020). True Event-Driven and Fault-Tolerant Routing in Wireless Sensor Network. Wirel. Pers. Commun..

[60] Muhammed T., Shaikh R.A. (2017). An analysis of fault detection strategies in wireless sensor networks. J. Netw. Comput. Appl..

[61] Shukry S. (2021). Stable routing and energy-conserved data transmission over wireless sensor networks. EURASIP J. Wirel. Commun. Netw..

[62] Singh J., Kaur R., Singh D. (2020). A survey and taxonomy on energy management schemes in wireless sensor networks. J. Syst. Arch..

[63] Abdul-Qawy A.S.H., Almurisi N.M.S., Tadisetty S. (2020). Classification of Energy Saving Techniques for IoT-based Heterogeneous Wireless Nodes. Procedia Comput. Sci..

[64] Nakas C., Kandris D., Visvardis G. (2020). Energy Efficient Routing in Wireless Sensor Networks: A Comprehensive Survey. Algorithms.

[65] Gaballah M., Alfadhli M., Abbod M. Network Structure Routing Protocols of WSN: Focus, Review & Analysis. Proceedings of the 2nd International Conference Broadband Communications, Wireless Sensors Powering, BCWSP 2020.

[66] Saraswathi S., Suresh G.R., Katiravan J. (2021). False alarm detection using dynamic threshold in medical wireless sensor networks. Wirel. Netw..

[67] Baniata M., Reda H.T., Chilamkurti N., Abuadbba A. (2021). Energy-Efficient Hybrid Routing Protocol for IoT Communication Systems in 5G and Beyond. Sensors.

[68] Chanak P., Banerjee I. (2016). Fuzzy rule-based faulty node classification and management scheme for large scale wireless sensor networks. Expert Syst. Appl..

[69] Yemeni Z., Wang H., Ismael W.M., Hawbani A., Chen Z. (2021). CFDDR: A Centralized Faulty Data Detection and Recovery Approach for WSN With Faults Identification. IEEE Syst. J..

[70] Chander B., Kumaravelan G. (2021). Outlier detection strategies for WSNs: A survey. J. King Saud Univ. Comput. Inf. Sci..

[71] Azzouz I., Boussaid B., Zouinkhi A., Abdelkrim M.N. Multi-faults classification in WSN: A deep learning approach. Proceedings of the International Conference on Sciences and Techniques of Automatic Control and Computer Engineering (STA).

[72] Tavallali P., Tavallali P., Singhal M. (2021). K-means tree: An optimal clustering tree for unsupervised learning. J. Supercomput..

[73] Lavanya S., Prasanth A., Jayachitra S., Shenbagarajan A. (2021). A Tuned Classification Approach for Efficient Heterogeneous Fault Diagnosis in IoT-enabled WSN Applications. Measurement.

[74] Rajan M.S., Dilip G., Kannan N., Namratha M., Majji S., Mohapatra S.K., Patnala T.R., Karanam S.R. (2021). Diagnosis of fault node in wireless sensor networks using adaptive neuro-fuzzy inference system. Appl. Nanosci..

[75] Yu T., Akhtar A.M., Wang X., Shami A. Temporal and spatial correlation based distributed fault detection in wireless sensor networks. Proceedings of the 28th Canadian Conference on Electrical and Computer Engineering (CCECE).

[76] Jin X., Chow T.W.S., Sun Y., Shan J., Lau B.C.P. (2015). Kuiper test and autoregressive model-based approach for wireless sensor network fault diagnosis. Wirel. Netw..

[77] Kaur R., Sandhu J.K., Sapra L. Machine Learning Technique for Wireless Sensor Networks. Proceedings of the International Conference on Parallel Distributed and Grid Computing (PDGC).

[78] Javaid A., Javaid N., Wadud Z., Saba T., Sheta O.E., Saleem M.Q., Alzahrani M.E. (2019). Machine Learning Algorithms and Fault Detection for Improved Belief Function Based Decision Fusion in Wireless Sensor Networks. Sensors.

[79] Sutagundar A.V., Bennur V.S., Anusha A., Bhanu K. Agent based fault tolerance in wireless sensor networks. Proceedings of the 2016 International Conference on Inventive Computation Technologies (ICICT).

[80] Qiu T., Chen N., Li K., Qiao D., Fu Z. (2017). Heterogeneous ad hoc networks: Architectures, advances and challenges. Ad Hoc Netw..

[81] Kiran W.S., Smys S., Bindhu V. (2021). Clustering of WSN Based on PSO with Fault Tolerance and Efficient Multidirectional Routing. Wirel. Pers. Commun..

[82] Habash M.Y., Ayad N.M.A.E., Ammar A.E.A.E. (2021). Fault tolerant radiation monitoring system using wireless sensor and actor network in a nuclear facility. Int. J. Electron. Telecommun..

[83] Saeed U., Jan S.U., Lee Y.-D., Koo I. (2021). Fault diagnosis based on extremely randomized trees in wireless sensor networks. Reliab. Eng. Syst. Saf..

[84] Belkadi K., Lehsaini M. Energy-efficient fault-tolerant routing for wireless sensor networks. Proceedings of the 2nd International Work, Human-Centric Smart Environ, Heal, Well-Being, IHSH 2020.

[85] Bhat S.J., Santhosh K.V. (2021). A Method for Fault Tolerant Localization of Heterogeneous Wireless Sensor Networks. IEEE Access.

[86] Liang W., Ma C., Zheng M., Luo L. (2021). Relay Node Placement in Wireless Sensor Networks: From Theory to Practice. IEEE Trans. Mob. Comput..

[87] Fu X., Wang Y., Li W., Yang Y., Postolache O. (2021). Lightweight Fault Detection Strategy for Wireless Sensor Networks Based on Trend Correlation. IEEE Access.

[88] Prikopa K.E., Gansterer W.N. (2020). Fault-tolerant least squares solvers for wireless sensor networks based on gossiping. J. Parallel Distrib. Comput..

[89] Moussa N., Alaoui A.E.B.E., Chaudet C. (2020). A novel approach of WSN routing protocols comparison for forest fire detection. Wirel. Netw..

[90] Priyadarshini R.R., Sivakumar N. (2020). Failure prediction, detection & recovery algorithms using MCMC in tree-based network topology to improve coverage and connectivity in 3D-UW environment. Appl. Acoust..

[91] Acharya S., Tripathy C. (2020). A reliable fault-tolerant ANFIS model based data aggregation scheme for Wireless Sensor Networks. J. King Saud Univ.-Comput. Inf. Sci..

[92] Arapoglu O., Dagdeviren O. (2021). A fault-tolerant and distributed capacitated connected dominating set algorithm for wireless sensor networks. Comput. Stand. Interfaces.

[93] Hmaid S.A.A.B., Vasanthi V. (2020). Fractional Gaussian Firefly Algorithm and Darwinian Chicken Swarm Optimization for IoT Multipath Fault-Tolerant Routing. Int. J. Comput. Netw. Appl..

[94] Alemayehu T.S., Kim J.-H., Yoon W. (2020). Fault-Tolerant UAV Data Acquisition Schemes. Wirel. Pers. Commun..

[95] Sivakumar S., Vivekanandan P. (2020). Efficient fault-tolerant routing in IoT wireless sensor networks based on path graph flow modeling with Marchenko–Pastur distribution (EFT-PMD). Wirel. Netw..

[96] Surya S., Ravi R. (2020). Concoction Node Fault Discovery (CNFD) on Wireless Sensor Network Using the Neighborhood Density Estimation in SHM. Wirel. Pers. Commun..

[97] Moridi E., Haghparast M., Hosseinzadeh M., Jassbi S.J. (2020). Novel fault-tolerant clustering-based multipath algorithm (FTCM) for wireless sensor networks. Telecommun. Syst..

[98] Pandey O.J., Gautam V., Nguyen H.H., Shukla M.K., Hegde R.M. (2020). Fault-Resilient Distributed Detection and Estimation Over a SW-WSN Using LCMV Beamforming. IEEE Trans. Netw. Serv. Manag..

[99] Swain R.R., Khilar P.M., Dash T. (2020). Multifault diagnosis in WSN using a hybrid metaheuristic trained neural network. Digit. Commun. Netw..

[100] Karmarkar A., Chanak P., Kumar N. An Optimized SVM based Fault Diagnosis Scheme for Wireless Sensor Networks. Proceedings of the 2020 IEEE International Students’ Conference on Electrical, Electronics and Computer Science (SCEECS).

[101] Mehmood G., Khan M.Z., Abbas S., Faisal M., Rahman H.U. (2020). An Energy-Efficient and Cooperative Fault- Tolerant Communication Approach for Wireless Body Area Network. IEEE Access.

[102] Bhat S.J., Santhosh K.V. Fault Tolerant Localization Based on K-means Clustering in Wireless Sensor Networks. Proceedings of the IEEE CONECCT 2020: 6th International Conference on Electronics, Computing and Communication Technologie.

[103] Jia S., Ma L., Qin D. (2019). Research on low energy consumption distributed fault detection mechanism in wireless sensor network. China Commun..

[104] Swain R.R., Dash T., Khilar P.M. (2019). A complete diagnosis of faulty sensor modules in a wireless sensor network. Ad Hoc Netw..

[105] Shen Z., Tagami A., Higashino T. An Efficient Data Processing Scheme for Wireless Sensor Network Monitoring Using a Machine Learning Model. Proceedings of the 2018 Eleventh International Conference on Mobile Computing and Ubiquitous Network (ICMU).

[106] Feiyue Y., Yang T., Siqing Z., Jianjian D., Juan X., Yao H. (2018). A Faulty Node Detection Algorithm based on Spatial-temporal Cooperation in Wireless Sensor Networks. Procedia Comput. Sci..

[107] Das Mohapatra A., Sahoo M.N., Sangaiah A.K. (2018). Distributed fault diagnosis with dynamic cluster-head and energy efficient dissemination model for smart city. Sustain. Cities Soc..

[108] Yuan Y., Li S., Zhang X., Sun J. A Comparative Analysis of SVM, Naive Bayes and GBDT for Data Faults Detection in WSNs. Proceedings of the IEEE 18th International Conference on Software Quality, Reliability, and Security Companion QRS-C.

[109] Shafeeq U., Chanak P. Heterogeneous Hardware Fault-Detection Scheme for Large Scale Wireless Sensor Networks. Proceedings of the 2018 International Conference on Circuits and Systems in Digital Enterprise Technology, ICCSDET 2018.

[110] Bhoi S.K., Obaidat M.S., Puthal D., Singh M., Hsiao K.-F. Software Defined Network Based Fault Detection in Industrial Wireless Sensor Networks. Proceedings of the 2018 IEEE Global Communications Conference (GLOBECOM).

[111] Swain R.R., Khilar P., Bhoi S.K. (2018). Heterogeneous fault diagnosis for wireless sensor networks. Ad Hoc Netw..

[112] Raja K., Leichombam R. Distributed Fault Node Detection and Classification using Fuzzy Logic and Management Scheme for Wireless Sensor Networks. Proceedings of the 2018 Tenth International Conference on Advanced Computing.

[113] Alshammari K., Ahmed A.E.S. An efficient approach for detecting nodes failures in wireless sensor network based on clustering. Proceedings of the International Symposium on Networks, Computers and Communications (ISNCC).

[114] Joshi V., Desai O., Kowli A. High accuracy sensor fault detection for energy management applications. Proceedings of the IEEE International Conference on Signal Processing, Informatics, Communication and Energy Systems (SPICES).

[115] Chanak P., Banerjee I., Sherratt R. (2016). Mobile sink based fault diagnosis scheme for wireless sensor networks. J. Syst. Softw..

[116] Swain R.R., Khilar P.M. A fuzzy MLP approach for fault diagnosis in wireless sensor networks. Proceedings of the IEEE Region 10 International Conference TENCON.

[117] Rajeswari K., Neduncheliyan S. Cluster based fault tolerance using genetic algorithm in wireless sensor network. Proceedings of the International Conference on Information Communication and Embedded Systems (ICICES).

[118] Benkaouha H., Abdelli A., Guerroumi M., Ben-Othman J., Mokdad L. EAFD, a failure detector for clustered WSN. Proceedings of the IEEE International Conference on Communications.

[119] Sutaone M., Mukherj P., Paranjape S. Trust-based Cluster head validation and outlier detection technique for Mobile Wireless Sensor Networks. Proceedings of the 2016 IEEE International Conference on Wireless Communications, Signal Processing and Networking, WiSPNET.

[120] Singh S.S., Jinila Y.B. Sensor node failure detection using check point recovery algorithm. Proceedings of the International Conference on Recent Trends in Information Technology (ICRTIT).

[121] Yu T., Wang X., Shami A. A novel R-PCA based multivariate fault-tolerant data aggregation algorithm in WSNs. Proceedings of the IEEE International Conference on Communications.

[122] Noshad Z., Javaid N., Saba T., Wadud Z., Saleem M.Q., Alzahrani M.E., Sheta O.E. (2019). Fault Detection in Wireless Sensor Networks through the Random Forest Classifier. Sensors.

[123] Fu X., Yao H., Yang Y. (2019). Modeling and Analyzing the Cascading Invulnerability of Wireless Sensor Networks. IEEE Sens. J..

[124] Xu P., Wu J., Chang C.-Y., Shang C., Roy D.S. (2021). MCDP: Maximizing Cooperative Detection Probability for Barrier Coverage in Rechargeable Wireless Sensor Networks. IEEE Sens. J..

[125] Baniabdelghany H., Obermaisser R., Khalifeh A. (2022). Reliable Task Allocation for Time-Triggered IoT-WSN using Discrete Particle Swarm Optimization. IEEE Int. Things J..

[126] Zhang Y., Wu H., Mei X., Liang L., Gulliver T.A. (2022). Unknown Transmit Power RSSD-Based Localization in a Gaussian Mixture Channel. IEEE Sens. J..

[127] Frohlich A.A., Scheffel R.M., Kozhaya D., Verissimo P.E. (2019). Byzantine Resilient Protocol for the IoT. IEEE Int. Things J..

[128] Zidi S., Moulahi T., Alaya B. (2018). Fault Detection in Wireless Sensor Networks Through SVM Classifier. IEEE Sens. J..

[129] Kaiwartya O., Abdullah A.H., Cao Y., Lloret J., Kumar S., Shah R.R., Prasad M., Prakash S. (2018). Virtualization in Wireless Sensor Networks: Fault Tolerant Embedding for Internet of Things. IEEE Int. Things J..

[130] Hasan M.Z., Al-Turjman F. (2017). Optimizing Multipath Routing With Guaranteed Fault Tolerance in Internet of Things. IEEE Sens. J..

[131] Thomas D., Orgun M., Hitchens M., Shankaran R., Mukhopadhyay S.C., Ni W. (2021). A Graph-Based Fault-Tolerant Approach to Modeling QoS for IoT-Based Surveillance Applications. IEEE Int. Things J..

[132] Tong Y., Tian L., Lin L., Wang Z. (2020). Fault Tolerance Mechanism Combining Static Backup and Dynamic Timing Monitoring for Cluster Heads. IEEE Access.

[133] Wang T., Zhang Y., Xiong N.N., Wan S., Shen S., Huang S. (2022). An Effective Edge-Intelligent Service Placement Technology for 5G-and-Beyond Industrial IoT. IEEE Trans. Ind. Inform..

[134] Feng S., Shi H., Huang L., Shen S., Yu S., Peng H., Wu C. (2021). Unknown hostile environment-oriented autonomous WSN deployment using a mobile robot. J. Netw. Comput. Appl..

[135] Shankar A., Sivakumar N.R., Sivaram M., Ambikapathy A., Nguyen T.K., Dhasarathan V. (2021). Increasing fault tolerance ability and network lifetime with clustered pollination in wireless sensor networks. J. Ambient Intell. Humaniz. Comput..

[136] Srivastava V., Tripathi S., Singh K., Son L.H. (2020). Energy efficient optimized rate based congestion control routing in wireless sensor network. J. Ambient Intell. Humaniz. Comput..

[137] Rehena Z., Mukherjee R., Roy S., Mukherjee N. Detection of node failure in Wireless Sensor Networks. Proceedings of the 2014 Applications and Innovations in Mobile Computing (AIMoC).

[138] Panda M., Khilar P. (2015). Distributed Byzantine fault detection technique in wireless sensor networks based on hypothesis testing. Comput. Electr. Eng..

[139] Lima M.M., Oliveira H.A., Guidoni D.L., Loureiro A.A. (2017). Geographic routing and hole bypass using long range sinks for wireless sensor networks. Ad Hoc Netw..

